# Trace compounds in Early Medieval Egyptian blue carry information on provenance, manufacture, application, and ageing

**DOI:** 10.1038/s41598-021-90759-6

**Published:** 2021-05-28

**Authors:** Petra Dariz, Thomas Schmid

**Affiliations:** 1 School of Analytical Sciences Adlershof (SALSA), Humboldt-Universität Zu Berlin, 10099 Berlin, Germany; 2 Bundesanstalt Für Materialforschung Und -Prüfung (BAM), 12489 Berlin, Germany

**Keywords:** Imaging studies, Mineralogy

## Abstract

Only a few scientific evidences for the use of Egyptian blue in Early Medieval wall paintings in Central and Southern Europe have been reported so far. The monochrome blue fragment discussed here belongs to the second church building of St. Peter above Gratsch (South Tyrol, Northern Italy, fifth/sixth century A.D.). Beyond cuprorivaite and carbon black (underpainting), 26 accessory minerals down to trace levels were detected by means of Raman microspectroscopy, providing unprecedented insights into the raw materials blend and conversion reactions during preparation, application, and ageing of the pigment. In conjunction with archaeological evidences for the manufacture of Egyptian blue in Cumae and Liternum and the concordant statements of the antique Roman writers Vitruvius and Pliny the Elder, natural impurities of the quartz sand speak for a pigment produced at the northern Phlegrean Fields (Campania, Southern Italy). Chalcocite (and chalcopyrite) suggest the use of a sulphidic copper ore, and water-insoluble salts a mixed-alkaline flux in the form of plant ash. Not fully reacted quartz crystals partly intergrown with cuprorivaite and only minimal traces of silicate glass portend solid-state reactions predominating the chemical reactions during synthesis, while the melting of the raw materials into glass most likely played a negligible role.

## Introduction

According to ancient Greek and Roman writers (Theophrastus, Vitruvius and Pliny the Elder), Egyptian blue, the first artificial pigment of mankind, was invented in Egypt, hence its name. The classical blue pigment of antiquity was used extensively and almost exclusively from the early dynasties in Egypt until the end of the Roman period in Europe. The natural analogue cuprorivaite CuCaSi_4_O_10_^1^, corresponding in its chemical and mineralogical properties, is too rare in nature for such a widespread utilisation. In the case of provincial Roman wall paintings, several scientific evidences for the application of Egyptian blue are published^2–6^, but findings on works from the Early Middle Ages in the same geographical context have only been sporadic, at least so far. This pronounced decline in use led to the hypothesis of an interrupted transfer of knowledge with regard to raw material blend and parameters of manufacture but might simply reflect the currently insufficient data situation. The occurrence of Egyptian blue on medieval murals is documented, for example, in San Saba in Rome (Lazio, Italy, first half of the eighth century A.D.) in a mixture with lapis lazuli^7^, in St. Benedict in Mals (South Tyrol, Italy, around 800 A.D.)^8^, in the monastery church and in the chapel of the Holy Cross of St. John in Müstair (Grisons, Switzerland, Carolingian and Romanesque wall paintings)^8, 9^, in the lower church of San Clemente in Rome (Lazio, Italy, middle of the ninth century A.D.)^10^, and in Santa Maria foris portas in Castelseprio (Lombardy, Italy, tenth century A.D.)^11^.

The blue pigment is synthesised by sintering or melting a raw material mixture of quartz sand or pebbles, limestone, flux in the form of either soda or ash from halophytes (salt plants), and a copper compound (copper mineral or possibly also alloy), according to modern laboratory experiments at temperatures between 850 °C and 1000 °C under oxidising conditions^12–18^. In the first century B.C. Vitruvius described background and procedure in his architectural textbook *De architectura libri decem* without any information on quantities and temperatures: “The recipes for blue [sky blue] were first discovered in Alexandria, and subsequently Vestorius began to manufacture it in Puteoli as well. Its story and how it was invented are quite marvelous. Sand is ground with flower of natron [first-class potassium nitrate] so finely that it almost becomes like flour. Copper, broken by coarse files until it is like sawdust, is sprinkled with this sand until it clings together. Then it is formed into balls by rolling it between the hands and bound together to dry. Once dry, the balls are put into a ceramic pitcher, and the pitchers are put into a kiln. In this way the copper and the sand, boiling with the energy of the fire, bond together, and exchanging their sweat between them they leave off their original properties, with their natures merged they produce a blue color.” (Liber VII, Caput XI)^19^. Limestone or another calcium source, essential for the production of Egyptian blue, is not mentioned, probably due to an unrecognised natural carbonate content of the beach sands along the coastline of the Gulf of Naples (Campania, Italy). Isidore of Seville specifies the recipe insofar as he speaks of “cyprium in fornace adustum” (“*cyprium* [copper ore] that has been heated in the furnace”) in his encyclopedia *Etymologiarum sive originum libri XX* (Liber XIX, Caput XVII) published around 630 A.D.^20, 21^. In contrast to copper carbonate ores (azurite Cu_3_(CO_3_)_2_(OH)_2_ or malachite Cu_2_(CO_3_)(OH)_2_), sulphidic copper ores such as chalcopyrite CuFeS_2_ and chalcocite Cu_2_S have to be roasted prior to reduction to metallic copper in order to remove the sulphur by oxidation into sulphur dioxide.

In view of archaeological evidence, the uniform size of around 15 to 30 mm in diameter of the pigment balls traded in Roman times, and the information given by Vitruvius and Pliny the Elder^22^, current research assumes a monopolised production site at the Gulf of Pozzuoli, whereas manufacture in Central Europe is excluded due to the lack of relevant knowledge^23–28^. Unique hypotheses on the local origin of Roman Egyptian blue found in the form of intact pigment balls and wall painting fragments at the settlement Magdalensberg (Carinthia, Austria), and as pigment balls in a villa rustica near Borg (Saarland, Germany)^29, 30^, have been disproven^31, 32^.

**Figure 1:**
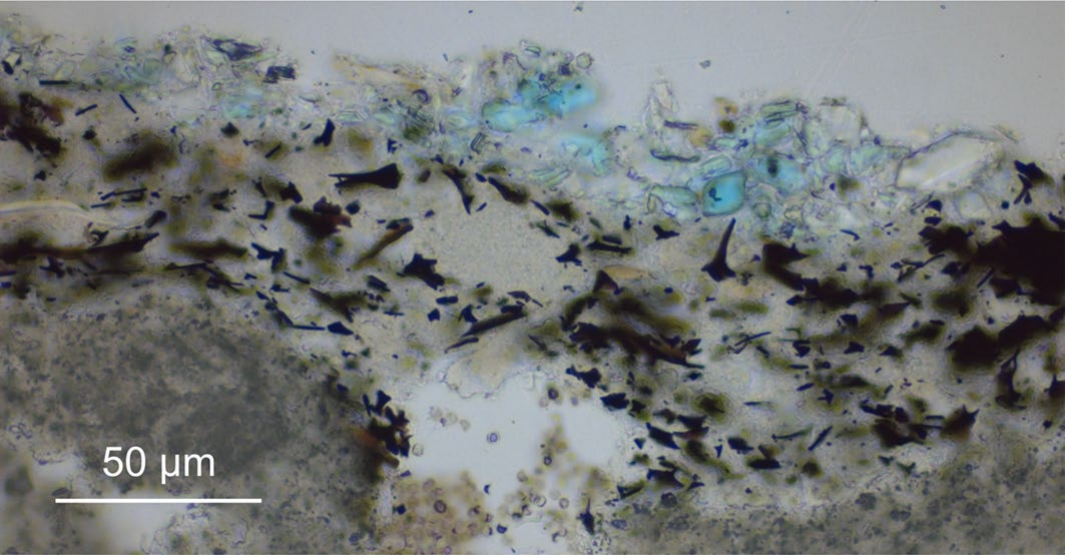
Micrograph of a polished thin section of the Egyptian blue paint layer and underpainting acquired with transmitted light and parallel Nicols.

After its rediscovery in the context of the Egyptian expedition and the excavations in Pompeii and Herculaneum, the elucidation of the chemical composition and structure of Egyptian blue were paramount, paralleled by laboratory experiments to determine the conditions required for an optimal pigment synthesis^33–38^. Only over the last decade, natural scientific research has pursued the characterisation and differentiation of production sites in the Mediterranean area on the basis of petrographic analyses of manufacturing remains^11, 23, 24, 39, 40^, but the analytical approaches undertaken so far did not reach beyond the main components (and only a few trace minerals) and were not able to provide quantitative information. The present study focuses on trace compounds capable of indicating the provenance of Egyptian blue found on a monochrome mural fragment excavated in the late 1970ies in the Langobardic church of St. Peter above Gratsch (South Tyrol, Northern Italy, first half of the eighth century A.D.). The remains of the wall painting have been assigned to the preceding major building phase dated by archaeologists before the Gothic War in 537–554 A.D.^41, 42^. As visible in Fig. 1, the blue pigment was applied on an underpainting consisting of lime-bound carbon black (plant black) and a not yet carbonated lime wash (‘fresco on lime wash’). Area-covering Raman microspectroscopic imaging^43^ with a spot size of around one micrometre enabled the identification of 26 minerals (beyond three main components, 23 traces at the sub-percent and sub-permille levels) further than cuprorivaite and carbon black, suggestive of type and source of the raw materials, and of chemical reactions occurring during pigment manufacture and application as well as ageing of the pictorial layer. Such individual insights into the plethora of phases and the specific history of the Egyptian blue in question represent a paradigm shift in the two-hundred-year research history^15, 44^.

## Methods

### Sample preparation

**Figure 2:**
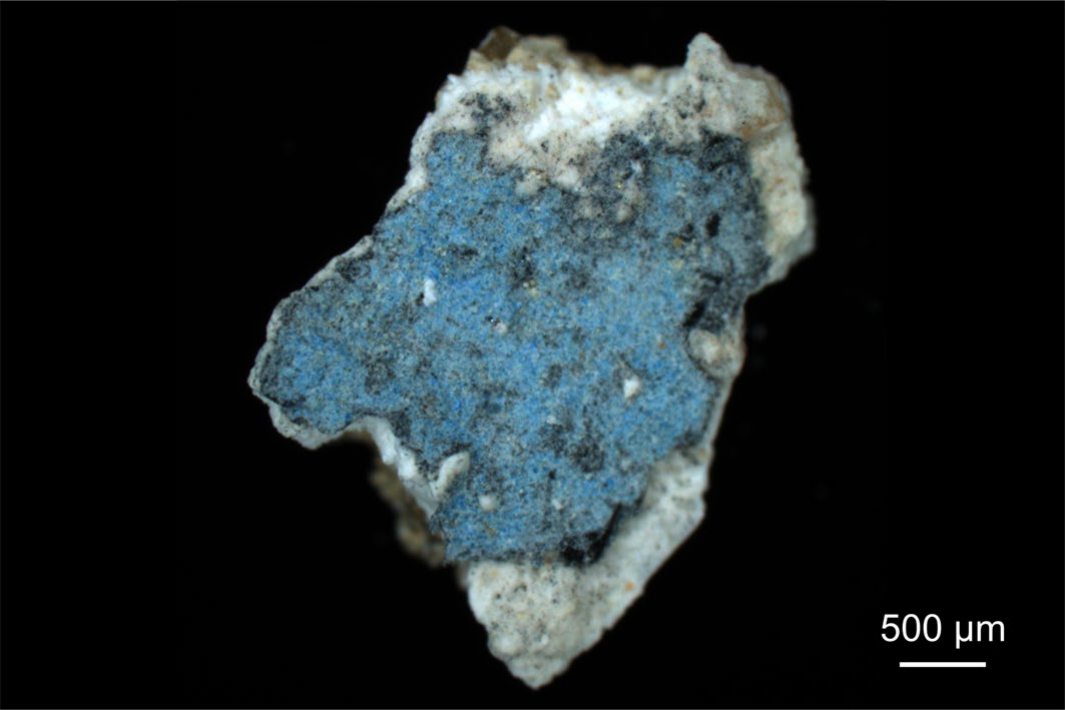
Darkfield light microscopy image of a patch of the pictorial layer of the mural fragment, consisting of Egyptian blue (on the top), grey underpainting, white lime wash and beige plaster.

Three patches of approx. 2–3 mm side length each (see Fig. 2) were removed from the Egyptian blue paint layer of the mural fragment by employing a scalpel. For Raman microspectroscopy, patches were placed on a microscope glass slide without further sample preparation. A resin-embedded polished thin section for light microscopy and a cross section were prepared in a specialised lab. For scanning electron microscopy, the cross section was coated with carbon and mounted onto a conductive sample holder by sticky carbon tape, while an accordingly mounted patch was imaged by environmental scanning electron microscopy at approx. 1 mbar without prior coating of its surface.

### Instrumentation

Raman spectra were acquired using a Horiba JobinYvon Labram HR800 Raman microscope with 532-nm continuous-wave laser excitation (diode-pumped solid-state laser, 40 mW maximum power at the sample surface). The laser light was focused onto the sample surface and the reflected or scattered light was collected in upright configuration by using a microscope objective. Dispersion of the Stokes-Raman-scattered light in an 800-mm spectrometer was carried out with a 300-mm^−1^ grating, and spectra were detected by a liquid-nitrogen cooled (− 130 °C operating temperature) charge coupled device (CCD) camera having 1024 pixels along the spectral axis. Raman maps were acquired by software-controlled (Horiba JobinYvon LabSpec 6) stepwise movement of the sample stage through the laser focus.

Light microscopy images were captured using a Zeiss AxioScope A.1 MAT with an AxioCam MRc Rev.3 camera, either in upright configuration and darkfield illumination, or in transmitted light with parallel Nicols. Scanning electron microscopy (SEM) was performed by employing a Jeol Neoscope JCM-6000 equipped with an energy-dispersive X-ray (EDX) module enabling high-vacuum (approx. 10^–4^ mbar) and low-vacuum (or environmental scanning electron microscopy) modes (approx. 1 mbar). Scanning electron micrographs were acquired by backscattered electron imaging (BSE or BEI, respectively).

### Conduction of Raman microspectroscopic analyses

Excitation at 532 nm wavelength enabled the acquisition of Raman spectra with strongly reduced disturbing autofluorescence emitted by the sample (as compared to excitation in the red spectral range at 632.8 nm or 785 nm also available with the employed instrument). By using a 50x/N.A. = 0.55 microscope objective (with N.A. denoting the numerical aperture), the theoretically calculated lateral focus diameter amounts to 1–1.2 µm (depending on the criterion used for its calculation; see Supplementary Information of Ref.^45^ for details), which is enlarged by light scattering on the corrugated surface of the paint layer under study. Thus, raster-scanning the sample in mapping experiments with a step size (or pixel size of the resulting Raman images, respectively) of 1 µm ensures full coverage of the sample surface while maintaining micrometric resolution in every measurement. The depth resolution of the microscope operated with open confocal pinhole (1 mm diameter) is estimated to approx. 40 µm in transparent samples^43^. By inserting a neutral density filter into the beam path, the laser power reaching the sample surface was reduced to 25%, i.e. 10 mW. That way, some crystalline phases sensitive to photo-induced or heat-induced conversions were kept in their original composition or oxidation state; for example, magnetite can be easily oxidised to hematite in Raman measurements using green laser excitation^46^. In each measurement spot, a Raman spectrum in the range of approx. 50 cm^−1^ to 3200 cm^−1^ was acquired with a wavenumber-dependent spectral resolution of 3.8 cm^−1^ to 2.6 cm^−1^ per CCD pixel as average of 10 consecutive acquisitions of 1 s each. The short acquisition time avoided saturation of the detector in almost all measurement spots of these highly heterogeneous samples, also in cases of strong autofluorescence emission. Averaging of 10 spectra not only improved the spectra quality in terms of signal-to-noise ratio but enabled partial bleaching of the autofluorescence emitted by the carbon black during laser exposure for 10 s.

In order to consider the heterogeneity of the samples, 21 Raman mapping experiments were performed on three different patches of the pictorial layer, one being shown by Fig. 2. The areas were randomly chosen and if possible, their size and shape were selected according to the sample’s topography to reduce the number of measurements in out-of-focus spots. The sizes of Raman maps ranged from 41 × 63 pixels (or micrometres, respectively) = 2,583 spectra to 101 × 155 pixels = 15,655 spectra. Altogether, 166,477 spectra were acquired and analysed. A typical experiment duration is one day and four hours as sum of the acquisition times of a 100 × 100 map plus the times needed for data read-out from the detector and stage movement. The mapping data were analysed using own LabVIEW-based (National Instruments Corp.) software, and Raman spectra were assigned to compounds by comparison with database spectra from the rruff spectral library^47^ or with literature data.

## Results and discussion

The observation of cuprorivaite by Raman microspectroscopy identified Egyptian blue as the colouring pigment of the monochrome blue paint layer (see Figs. S1-S6 in the Supplementary Information). As a micrometrically ground, heterogeneous assemblage, the examined synthesised pigment depicts the mineralogy of the raw materials and includes indicators for transformation reactions during production, application and ageing. The large variety in area coverage fractions from percent down to sub-permille levels (see Table S1 in the Supplementary Information for details on the quantification approach) and the sizes of the crystalline components in the lower micrometre range pose challenges for the read-out of these information carriers. For example, the identification of trace indicator minerals in bulk analyses by powder X-ray diffraction is impossible due to the detection limit of a few percent^25, 48^. Electron microprobe enables the quantitative determination of local elemental compositions and their distributions in carbon-coated polished thin sections of mortar or pictorial layer samples with a very high spatial resolution in the sub-micrometre range, but is limited to pure elemental analysis and incapable to discern low-atomic-number elements. As a combination of imaging and molecular spectroscopy, Raman microspectroscopy provides a resolution in the range of a few hundred nanometres^43^ and thus permits the precise differentiation of neighbouring crystals within the microscopically structured paint layer, if performed as area-covering imaging experiment.

Within the present study, non-destructive Raman microspectroscopic measurements of the patches of the blue paint layer were carried out without any sample preparation (see Methods section for details). Green laser excitation at 532 nm was well suited to detect Raman spectra with only weak interference by autofluorescence emitted by the material: orange-red fluorescence of the carbon black pigment and the well-known near-infrared luminescence of cuprorivaite (see Figs. S2 and S4 in the Supporting Information)^28, 49–54^. As explained in the Methods section, 166′477 Raman spectra were acquired in total, which were assigned to one or a superposition of a few crystalline or amorphous phases constituting the pictorial layer by matching with reference data from either a spectral library^47^ or the literature (see Figs. S7–S20 in the Supplementary Information for details on the spectral assignments). This laborious method provided access to 28 different compounds, which are listed in Table 1 in decreasing order of their frequency of occurrence.

**Table 1:** Minerals identified in the paint layer by Raman microspectroscopy.

Mineral phase	Formula	Source/interpretation
** > 1% of the assigned Raman spectra:**		
Cuprorivaite	CaCuSi_4_O_10_	Synthesis
Calcite	CaCO_3_	Quartz sand/carbonation
Amorphous carbon	C	Underpainting
Quartz	SiO_2_	Quartz sand
Feldspars	M(I)_x_M(II)_1-x_Al_2-x_Si_2+x_O_8_	Quartz sand
** < 1% of the assigned Raman spectra:**		
Syngenite	K_2_Ca(SO_4_)_2_·H_2_O	Application (plant ash)
Weddellite (oxalate)	Ca(C_2_O_4_)·2H_2_O	Ageing (microorganisms)
Augite-diopside	(Ca,Mg,Fe)_2_Si_2_O_6_	Quartz sand
Aegirine	NaFeSi_2_O_6_	Quartz sand
Chalcocite	Cu_2_S	Copper ore
Natrojarosite	NaFe_3_(SO_4_)_2_(OH)_6_	Copper ore
Basic copper arsenate	Cu_x_(AsO_4_)_y_(OH)_2x-3y_	Copper ore
Cassiterite	SnO_2_	Copper ore
Magnetite	Fe_3_O_4_	Quartz sand/copper ore
** ≤ 1‰ of the assigned Raman spectra:**		
Cristobalite	SiO_2_	Quartz sand/synthesis (?)
Apatite	Ca_5_(PO_4_)_3_(F,OH)	Quartz sand/copper ore/synthesis
Osarizawaite	PbCuAl_2_(SO_4_)_2_(OH)_6_	Copper ore
Eskolaite	Cr_2_O_3_	Copper ore
Malayaite	CaSnOSiO_4_	Synthesis (copper ore)
Polyhalite	K_2_Ca_2_Mg(SO_4_)_4_·2H_2_O	Application (plant ash)
Anatase	TiO_2_	Quartz sand/copper ore/synthesis
Copper oxide	Cu_x_O	Synthesis (copper ore)
Aragonite	CaCO_3_	Carbonation
Lead oxide	PbO_x_	Synthesis (copper ore)
Dolomite	CaMg(CO_3_)_2_	Quartz sand
Jacobsite	MnFe_2_O_4_	Copper ore
Hematite	Fe_2_O_3_	Quartz sand/synthesis
Silicate glass	“SiO_2_”	Synthesis (quartz sand)

### Mineralogy of the quartz sand

**Figure 3:**
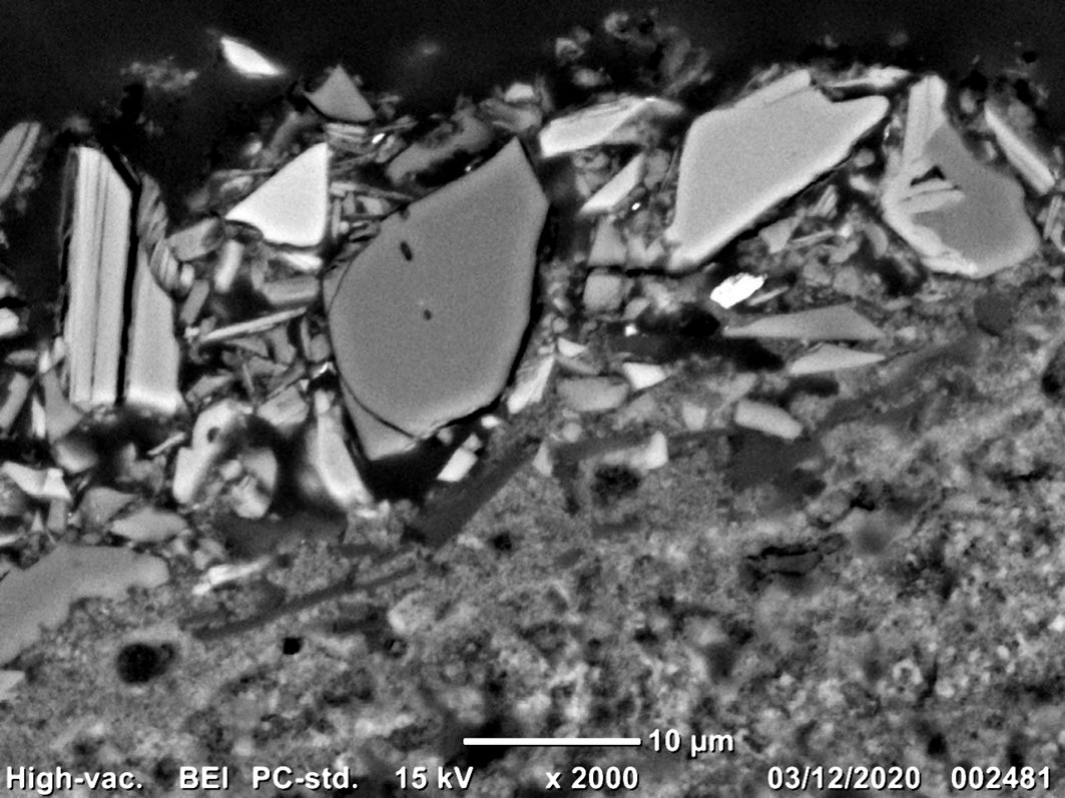
Scanning electron micrograph of a cross-sectional sample of the pictorial layer acquired in backscattered electron imaging mode (BEI). As elements with high atomic numbers cause strong electron backscattering, the brightest particles can be assigned to copper oxides (confirmed by energy dispersive X-ray spectroscopy, data not shown), followed by cuprorivaite appearing slightly darker. The grain on the top right consists of intergrown cuprorivaite and quartz. The barely visible silicate layers in the blue pigment are much more pronounced in micrometric to sub-micrometric particles seen on the left.

As can be seen in the SEM-BSE micrograph of the Egyptian blue paint layer in Fig. 3, some quartz grains are intergrown with cuprorivaite. Relictic or recrystallised α-quartz in conjunction with largely missing high-temperature phases such as wollastonite CaSiO_3_, gehlenite Ca_2_Al(AlSi)O_7_ as well as the two high-temperature polymorphs of SiO_2_ tridymite (transformation temperature 870 °C) and cristobalite (1470 °C) speak for process temperatures below 900 °C. Seen the decomposition temperature of cuprorivaite of approx. 1050 °C^18^, the few cristobalite grains in the paint layer (detected in 0.1% of all spectra) are most likely remnants of the quartz sand. Unlike tridymite and cristobalite, quartz can only form within its stability field, that is, when crystallising from a melt, its temperature cannot have been significantly higher than 870 °C. Trigonal α-quartz reversibly converts into the high-temperature β-polymorph at 573 °C; however, the β-quartz structure of hexagonal symmetry cannot be stabilised by quenching, which is why this transition is not suitable as a mineral thermometer^55^.

**Figure 4:**
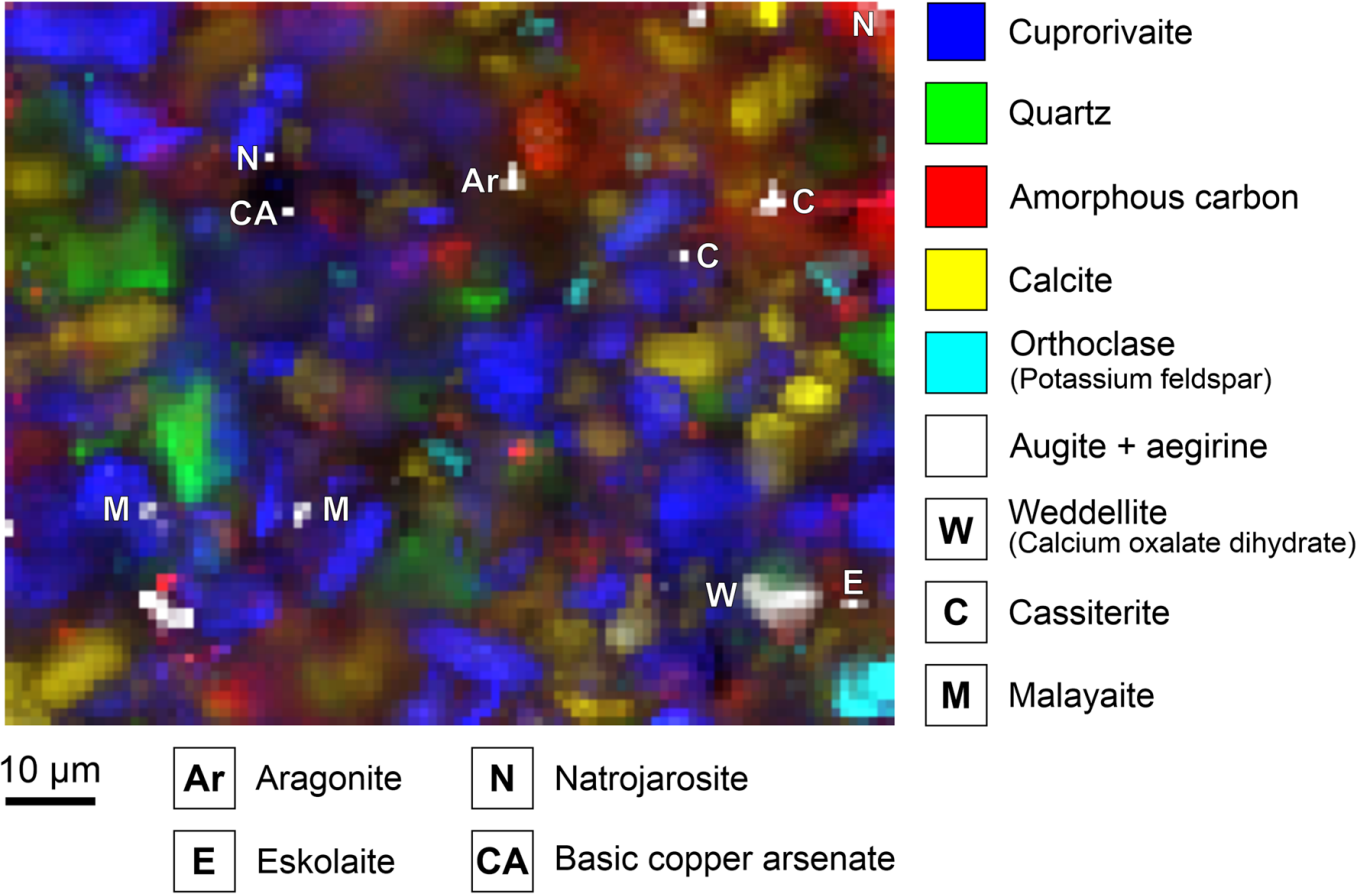
Raman map of the Egyptian blue paint layer. The intensities of marker bands reveal the distributions of eight trace constituents beyond the main components cuprorivaite, amorphous carbon (carbon black in the underpainting), quartz, calcite, and the feldspar orthoclase.

The Raman microspectroscopic analyses of the Egyptian blue (see an example in Fig. 4) reveal calcite CaCO_3_ in different grain sizes. The sparitic crystals are probably not completely decarbonated limestone fragments from the raw material mixture or secondary formations as a result of recarbonation^56, 57^. In addition, due to the fragmentary state of preservation of the blue paint layer, the micritic lime binder of the underpainting—including the CaCO_3_ polymorph aragonite—is detected. The dolomite grains CaMg(CO_3_)_2_ can only be explained as remnant impurities from the quartz sand, not converted due to insufficient local temperatures. Thermal decomposition of dolomite into periclase MgO and calcium oxide CaO takes place in two steps, and the resulting calcium and magnesium components hydrate and carbonate separately from one another in terms of time and space to form calcite and magnesite^58–64^.

Further accessories of the processed quartz sand are preserved in the form of the two feldspars albite NaAlSi_3_O_8_ and orthoclase KAlSi_3_O_8_, as well as feldspar solid solutions (alkali and plagioclase feldspars). The evidence of celsian BaAl_2_Si_2_O_8_, belonging to the subgroup of the rare barium feldspars, should be emphasised here (see Figs. S7 and S8 in the Supplementary Information). In addition to these tectosilicates the mineral assemblage also include chain silicates, namely the two clinopyroxenes aegirine NaFeSi_2_O_6_ and augite (Ca, Na)(Mg, Fe, Al, Ti)(Si, Al)_2_O_6_ or their solid solution. (The typical zoning—augite in the core with a rim of aegirinaugite—cannot be observed due to the small particle size.) The monoclinic calcium pyroxene diopside CaMgSi_2_O_6_ also observed in the sample material forms a complete solid solution series with augite and hedenbergite CaFeSi_2_O_6_. As the reference spectra available in spectral libraries so far do not allow an unambiguous differentiation, these compounds are subsumed under ‘augite’ in Table 1. According to the experiments conducted by N. Bowen and J. Schairer, aegirine melts incongruously at 990 °C yielding hematite α-Fe_2_O_3_ (as well as magnetite Fe_3_O_4_) and melt; other phases in a multi-component system can increase or decrease this melting temperature^65^. The stability field of diopside lies above 1000 °C and thus likewise above the optimal process temperatures for the production of Egyptian blue. A pyrometamorphic genesis of diopside in the course of the pigment synthesis at the interface between quartz and dolomite grains at around 800 °C^62, 66^ appears unlikely in view of the few magnesium-containing phases detected in the pictorial layer.

The two iron oxides magnetite and hematite might be impurities of the quartz sand as well, but can also be seen in conjunction with the copper ore employed for the preparation of Egyptian blue, as hematite might result from pyrometamorphic conversions of accessory magnetite, pyrite FeS_2_ or secondary limonite FeO(OH)·nH_2_O. The low-temperature TiO_2_ polymorph anatase was observed as individual particles and as inclusions in quartz or cuprorivaite, at least some in nanometric sizes (see Fig. S18)^67^. At around 950 °C anatase is converted into the high-temperature modification rutile. As this polymorphic transformation strongly depends on parameters further than temperature, the suitability of the TiO_2_ system as a mineral thermometer is critically discussed in the literature^68, 69^. In addition to a natural TiO_2_ contamination of the quartz sand, other accessories such as ilmenite FeTiO_3_, titanomagnetite Fe(Fe,Ti)_2_O_4_ or sphene CaTiSiO_5_ as starting materials of TiO_2_ formation during pigment manufacture provide alternative hypotheses for the presence of anatase within the paint layer^27, 70^. Again, the copper ore cannot be ruled out as the source of this mineral. The mentioned hypotheses also apply to a member of the apatite family (fluoroapatite Ca_5_(PO_4_)_3_F, naturally predominantly occurring, rare hydroxyl apatite Ca_5_(PO_4_)_3_(OH), etc.), whose exact stoichiometry could not be determined based on the reference spectra available so far (see Fig. S13)^71^.

### Constituents and accessory minerals of the copper ore

The mineral chalcocite (copper glance) Cu_2_S identified in the paint layer by Raman microspectroscopy is indicative of the nature of the copper component in the raw materials blend used for the preparation of Egyptian blue. The presence of chalcopyrite CuFeS_2_ cannot be excluded due to the possibility of its conversion into Cu_2_S through the influence of the laser focus during Raman experiments^72^. In consistency with other studies, the pigment in question also contains the unreacted copper oxides tenorite CuO and cuprite Cu_2_O^12, 24, 27, 39, 40^. The according spectra match the Raman signatures of amorphous (or nanocrystalline) copper oxide mixtures Cu_x_O described in the literature (see Fig. S17)^73^. Tenorite and cuprite are typical oxidation products of sulphidic copper ores and of elemental copper, thus secondary minerals; on the other hand, both copper oxides can also be interpreted as evidence for roasting of the sulphidic copper ore prior to further processing and/or pigment synthesis in an oxidising furnace atmosphere.

Cassiterite (tin stone) SnO_2_ and malayaite CaSnOSiO_4_ (see Fig. S12) suggest an association of copper and tin ores, for example of the minerals chalcopyrite and stannite Cu_2_FeSnS_4_, typically along with sphalerite (zinc blende) ZnS, pyrite and galena (lead glance) PbS. Depending on the concentration, tin or tin-containing phases are interpreted as indicators for the recycling of bronze in several studies^27, 74^. For example, due to inclusions of cassiterite in cuprorivaite and in the glass phase, Gareth Hatton et al. assume bronze shavings as ingredients of the raw material mixture of Egyptian blue from Egypt and Mesopotamia^27^, idem Heiner Jaksch et al.^70^, Ioanna Kakoulli (Greek mural paintings)^75^, Celestino Grifa et al. (Egyptian blue incrustations on crucible fragments from Cumae, Campania, Italy)^24^ or Sandrine Pagès-Camagna and Sylvie Colinart (pigment cake and painted Egyptian green from Egypt)^13^. Incrustations of Egyptian blue on crucible fragments from Liternum (Campania, Italy) from the first century A.D. are tin-free^23^, this in accordance with wall painting samples from Pompeii also dated to the first century A.D.^27, 76^. Egyptian blue from fragments of a Carnuntine mural (Lower Austria) also contains no tin oxide, which Johannes Weber and Tatjana Bayerova justify with the melting of pure copper shavings^4^. In the case discussed here, the observation of tin minerals in the per mille range (both, cassiterite and malayaite were detected in approx. 0.1% of all assigned spectra) speaks against the processing of a copper-tin alloy or bronze, respectively.

**Figure 5:**
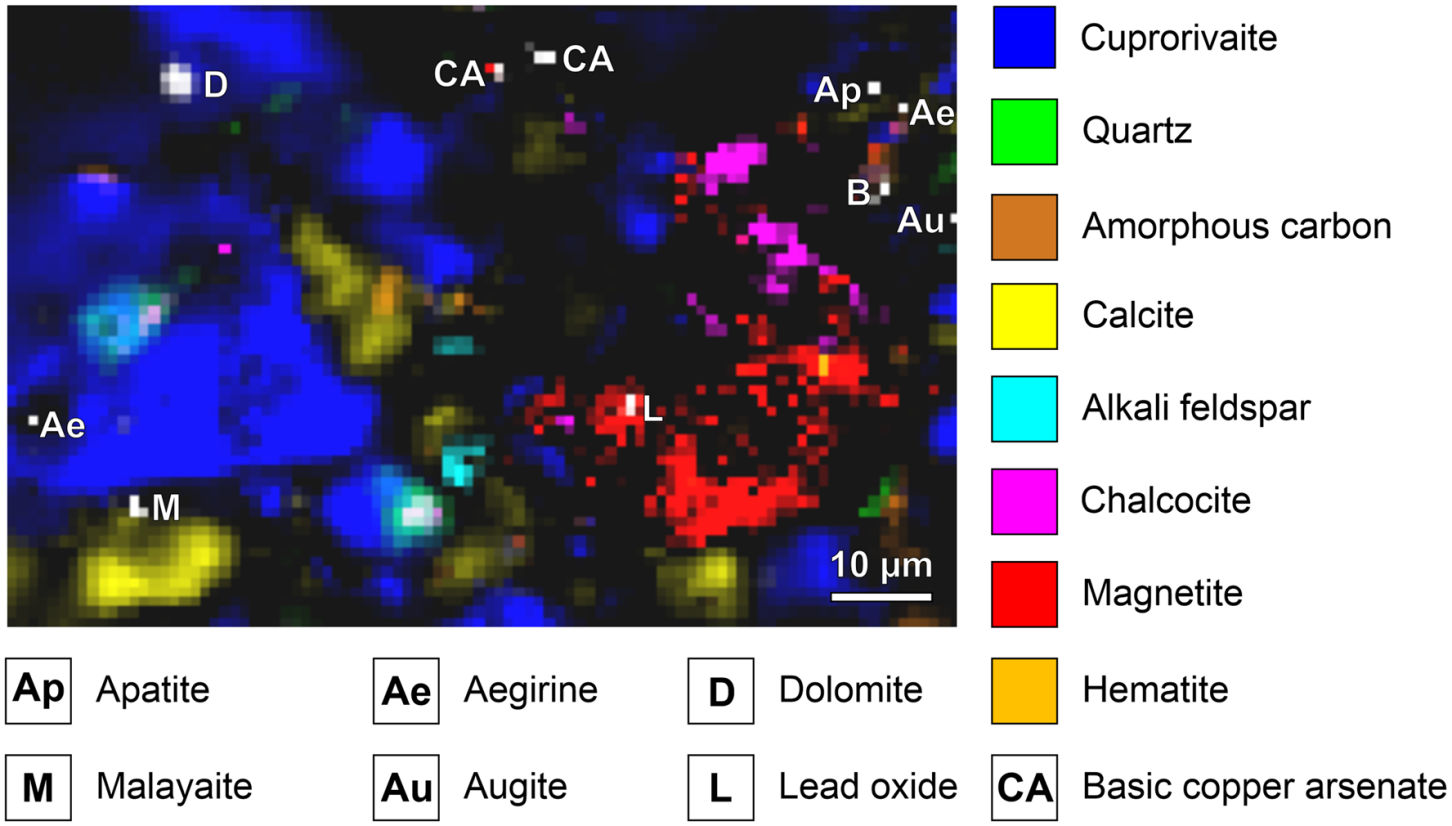
Raman map of the Egyptian blue paint layer. A remnant of the copper ore used as starting material in the pigment synthesis consists of chalcocite (and/or chalcopyrite) intergrown with magnetite. Hematite, basic copper arsenate, lead oxide and (perhaps) anatase can be interpreted as accessory minerals of the copper ore too.

Eskolaite Cr_2_O_3_ as well as the spinels magnetite (partially intergrown with chalcocite, see Fig. 5) and jacobsite MnFe_2_O_4_ are primary accessory minerals from the copper ore. Contrarily, the dimorphic basic copper arsenates clinoclase/gilmarite Cu_3_(AsO_4_)(OH)_3_ und cornubite/cornwallite Cu_5_(AsO_4_)_2_(OH)_4_ are commonly found as secondary minerals in the oxidation zone of hydrothermal copper deposits. Due to the ambiguous assignment of the experimental spectra to existing reference data (see Fig. S14)^77–79^, we generally term these minerals basic copper arsenates with the sum formula Cu_x_(AsO_4_)_y_(OH)_2x-3y_. In line with our findings, an unspecified copper arsenate mineral was spotted by means of Raman spectroscopy (main peak at 861 cm^−1^) in green powder from a pigment pot excavated at Pompeii archaeological site, a multicomponent mixture including (without indications of quantity) malachite, goethite, hematite, calcite, quartz, albite, cerussite PbCO_3_, massicot PbO and Egyptian blue^80^. Irene Aliatis et al. link the presence of copper arsenate to the exploited deposit carrying copper ores, ruling out the possibility of degradation of arsenical copper residues in the glass phase of Egyptian blue as stated by Ahmed El Goresy with regard to pharaonic wall paintings^81^.

The two sulphates osarizawaite PbCuAl_2_(SO_4_)_2_(OH)_6_ and natrojarosite NaFe_3_(SO_4_)_2_(OH)_6_ (see Fig. S16) can also be classified as oxidation products from a sulphidic copper ore, i.e. of accessory galena or pyrite, respectively. Due to the small amount (less than 0.2% of all assigned spectra) the intended use of natrojarosite as a yellow pigment can be excluded. The thermal degradation of natrojarosite comprises several steps, which are associated with inconsistent temperature specifications in different studies. According to the thermogravimetric data by Lei Chen, dehydroxilation yielding NaFe(SO_4_)_2_ and hematite at 447.5 °C is followed by desulphonation reactions at 682.6 °C and 716.5 °C resulting in Na_2_SO_4_, hematite and SO_3_^82^. Thus, the temperatures needed to prepare Egyptian blue exceed the stability range of natrojarosite, which like ossarizawaite (dehydration at 460 °C, desulphonation at 760 °C^83^) must have survived in colder zones in the clay pots or balls mentioned by Vitruvius, as mixing with water during application of the pigment might inverse a loss of crystal water, while the other decomposition reactions are irreversible in this respect.

Accessory galena can be oxidised to anglesite PbSO_4_ and finally be decomposed into plattnerite PbO_2_ during roasting of the sulphidic copper ore. The exact stoichiometry of the lead oxide compounds in the paint layer cannot be determined by Raman spectroscopy, as plattnerite is known to easily decompose into massicot because of the action of the laser employed in a Raman experiment (see Fig. S10)^84^. This reason for the observation of massicot together with its trace amount (only in 0.03% of all assigned spectra) rules out its intentional addition as yellow lead oxide pigment.

### Evidences for the flux used in the synthesis of the pigment

Virtually no evidence for the presence of a glass phase was found, as only about 0.01% of the spectra were unambiguously assigned to silicate glass^85–87^. Causes for this are probably the prevailing process parameters (synthesis temperature and regime, locally too low temperature or too short reaction time for homogeneous melt formation) and the composition of the mixture of starting materials (low flux content, no significant SiO_2_ excess) and not the loss of the glass phase by chemical weathering, as often assumed in studies dedicated to Egyptian blue^16, 29, 88^. There is some controversy on the theory of frit formation, initially favoured after the rediscovery of the blue pigment in the nineteenth century; while, for example, François Delamare assumes solid-state sintering with low and melt formation resulting in a glass fraction only with high alkali content^89, 90^, Trinitat Pradell et al. hypothesise nucleation and crystallisation of cuprorivaite from the cooling silicate melt regardless of the amount of flux in the raw material blend^16^. At least on laboratory scale, cuprorivaite can be synthesised through pure solid-state reactions, which requires the largest possible contact surfaces achieved by extensive homogenisation of the starting materials^91, 92^. Thus, the size of cuprorivaite crystals and grains of unreacted accessory minerals in the lower micrometre range observed in this study support our hypothesis of predominating solid-state reactions during pigment synthesis with negligible melt formation.

Usually, the ratios of Na_2_O/K_2_O and Na_2_O/MgO determined in Egyptian blue samples by means of elemental analysis (and recalculated into oxidic form according to convention) are employed to identify the type of flux in the raw material mixture, as they are affected by impurities in either soda or the ashes of halophytes. Both values are significantly lower in plant ashes, although potassium from natural impurities in the quartz sand (alkali feldspar, etc.) can also influence the concentration ratios of the chemical elements and thus lead to incorrect conclusions^13, 17, 93, 94^. Using Raman microscopy, only a few minerals containing magnesium were detected in the blue paint layer, potassium salts predominate, sodium is only present in albite and aegirine, but both are primary components of the quartz sand and thus not pyrometamorphic reaction products. The hydrous sulphate syngenite K_2_Ca(SO_4_)_2_·H_2_O is a secondary formation due to the aqueous suspension of the blue pigment, most probably a precipitate due to the reaction of arcanite K_2_SO_4_ from the plant ash with solvated calcium ions. (If there is no reduction to sulphite or sulphide, the thermal decomposition of arcanite only takes place above the stability field of cuprorivaite^93–96^.) The salt polyhalite K_2_Ca_2_Mg(SO_4_)_4_·2H_2_O or locally observed mixtures with syngenite have the same origin, possibly with langbeinite K_2_Mg_2_(SO_4_)_3_ or calciolangbeinite K_2_Ca_2_(SO_4_)_3_ as starting phase (see Fig. S15). The non-Raman-active salts with cubic structure sylvine KCl and halite NaCl were identified using the EDX detector of a scanning electron microscope (see Fig. S21).

This composition known in the field of glass research as "low magnesium, high potassium", suggests the use of a mixed alkali flux, for example in the form of the ashes of salt plants of the genera *Salsola* or *Suaeda* (both belonging to the saltworts) from the amaranth family (Amaranthaceae), which includes the glasswort *Salsola kali* (or synonym *Kali turgidum*) flourishing on the Mediterranean coasts^93, 97^. During glass production, sulphates and chlorides from the plant ash form a separate salt melt, the so called galle, whereas more reactive (hydrogen) carbonates, sulphites, sulphides and hydroxides are more easily absorbed into the molten glass^95^. An ion exchange between coexisting salt and silicate melts and analogous processes in the case of melt formation during the synthesis of Egyptian blue can be assumed, but in contrast to the manufacture of glass, the separation of unreacted salts is not part of the procedure described by Vitruvius. It is therefore possible that the ground blue pigment was washed before use in order to remove at least the easily soluble compounds. In the specific case of the Egyptian blue from the church of St. Peter, the vast absence of water-soluble salts such as mercallite KHSO_4_ might be explained as well by water contact during burial or wet cleaning after excavation.

### Ageing of the paint layer

The calcium oxalate dihydrate weddelite Ca(C_2_O_4_)·2H_2_O—whewellite Ca(C_2_O_4_)·H_2_O and glushinskite Mg(C_2_O_4_)·2H_2_O can be excluded due to the characteristic Raman signature—is a usual main component of patina layers on architectural heritage. In view of the only punctual occurrence of this alkaline earth oxalate on the surface of the wall painting fragment, the source of the oxalate anion is most likely the metabolism of microorganisms, while the oxidative degradation of historical organic paints and preservatives (no further organic compounds were detected), atmospheric deposition of oxalic acid H_2_C_2_O_4_, or calcium oxalate formed with calcitic fine dust appear unlikely, as there is sufficient calcite as a reactive starting material distributed throughout the pictorial layer. No evidences for the formation of heavy metal oxalates such as the copper salt moolooite Cu(C_2_O_4_)·0.5H_2_O as a result of the degradation of the artificial blue pigment by oxalic acid were found^54, 98, 99^. Some spectral signatures deviating from published references are therefore probably due to the influence of metal oxalates, whose spectra have not been described in the literature so far, or can be explained by the presence of salts of other short-chain (di)carboxylic acids, which can also result from the metabolism of microorganisms (see Fig. S20).

### Evidences for the provenance of the raw materials and the Egyptian blue pigment, respectively

Recently, petrographic investigations of the ceramic body of crucible fragments covered with residues of Egyptian blue frit, unearthed in the frame of archaeological campaigns in the ancient cities of Cumae and Literum (Campania, Italy), have been discussed by Celestino Grifa et al.^24^ as well as Lorenzo Lazzarini and Marco Verità^23^ in comparison with the mineralogical composition of local carbonate-bearing coastal sands, i.e. sediments transported by the Volturno river into the Gulf of Gaeta. As already published repeatedly, the “sand on a coast of six miles in length between Cumae and Liternum”, mentioned as suitable raw material for glass production by Pliny the Elder in his encyclopedia *Naturalis historiae* (Liber XXXVI, Caput XXVI or §194)^100^ in 77 A.D., contains impurities in the form of feldspars (potassium feldspar, hyalophanes (K,Ba)Al(Si,Al)_3_O_8_, albite, plagioclase), iron-rich augite, diopside (or iron-containing salite Ca(Mg,Fe)Si_2_O_6_), hornblende and volcanic rock fragments, as well as natural glass and sporadic accessories such as apatite, biotite K(Mg,Fe)_3_(Si_3_Al)O_10_(OH,F)_2_, rutile, ilmenite, sphene, garnet, magnetite, hematite, spinel and zircon ZrSiO_4_. Carbonates are primarily present as calcite, but also include dolomite; source rocks are the carbonate/siliciclastic successions of the Apennine chain^96, 101–105^.

Thus, with regard to the question of whether the Egyptian blue discussed here is imported from the northern Phlegrean Fields or manufactured locally in South Tyrol, the accessory minerals attributable to the quartz sand used (see detailed discussion above) embody relevant indications, in particular the clinopyroxenes aegirine and augite associated with volcanic activity and the seldom barium-rich alkali feldspar celsian. While carbonate-bearing quartz sand with a matching petrographic composition is not to be found in South Tyrol, sulphidic copper ore deposits are not uncommon. (In addition to the exploitation of the regionally most important chalcopyrite deposits from Prettau in the Ahrntal (Val Aurina) and on the Pfunderer Berg near Klausen (Chiusa), smaller-scale copper mining was carried out in the Stilfs-Trafoi area in the Vinschgau (Val Venosta) and in the Martell valley (Val Martello) on the slopes of the ridge between the Madritsch and Pedertal^106–108^.)

A potential comparison of the results presented here with corresponding blue pigments from Late Antique and Medieval wall paintings in South Tyrol, for example the Carolingian murals in the church of St. Benedict in Mals^8^, appears worthwhile for a sound scientific evidence of a production and trade monopoly at the Gulf of Pozzuoli surviving over centuries in the politically turbulent period after the fall of the Western Roman Empire.

## Conclusions

Only a few scientific evidences for the use of Egyptian blue, the classic blue pigment of Roman antiquity, in Early Medieval murals in Central and Southern Europe have been recorded so far. The monochrome blue fragment of a ‘fresco on lime wash’ discussed here, dated to fifth/sixth century A.D., belongs to the second church building of St. Peter above Gratsch (South Tyrol, Northern Italy). Beyond synthetic cuprorivaite (and carbon black from the underpainting), extensive raster-scanning of the surfaces of patches of the blue paint layer by means of Raman microspectroscopy was able to detect an assemblage of 26 accessory minerals down to the trace level representing valuable information carriers for provenancing the Egyptian blue in question. This astonishing diversity is only accessible by the analytical approach of an area-covering microspectroscopic imaging with high spatial resolution. Thus, the presented deep insights into the mineralogy of the raw materials and into conversion reactions during manufacture, application (water-insoluble salts formed out of the sulphate, alkali and alkaline earth metal ions of the flux), and ageing (oxalates as a result of microbial growth) of the artificial blue pigment are linked to technical advancements of the analytical sciences. Beyond the highly specific qualitative information enabling phase identification, the quantification of assigned Raman spectra provides access to the approximate area fraction covered by a mineral, which is prerequisite for its classification as either accessory mineral associated with the raw material blend surviving processing or in individual cases as intentionally added pigment.

Indicators for the production site in the form of natural impurities of the quartz sand deserve to be particularly emphasised: augite and aegirine, for example, suggest sediments exposed to magmatic activity (or volcanic activity related to the corresponding source area). According to literature, these two clinopyroxenes are in company with diopside and feldspars, including the rare barium-rich alkali feldspar celsian, characteristic components of carbonate-bearing sediments transported by the Volturno River into the Gulf of Gaeta. In conjunction with archaeological evidences for the manufacture of Egyptian blue in Cumae and Liternum (first century A.D.) and the complementary remarks of the two Roman writers Vitruvius and Pliny the Elder concerning workshops at Pozzuoli, this speaks for a pigment produced at the northern Phlegrean Fields (Campania, Southern Italy). In view of the detection of chalcocite (and chalcopyrite), a sulphidic copper ore—necessarily roasted to yield copper oxide—was presumably used as copper source. Water-insoluble salts like the hydrated sulphates syngenite and polyhalite or the alkali chlorides sylvine and halite imply the addition of a mixed-alkaline flux, i.e. a mixture of sodium and potassium salts in the form of plant ash. Not fully reacted quartz crystals partly intergrown with cuprorivaite and only minimal traces of silicate glass enable to deduce that solid-state reactions predominated the chemical reactions during pigment synthesis; the melting of the raw materials into glass most likely played a negligible role.

## Supplementary Information

Supplementary Information.

## Data Availability

The datasets generated during the current study are available from the corresponding author on reasonable request.
