# Supplementary material for: Trace compounds in Early Medieval Egyptian blue carry information on provenance, manufacture, application, and ageing
Source: Sci Rep. 2021 May 28;11:11296. doi: 10.1038/s41598-021-90759-6 (PMC8163881; doi:10.1038/s41598-021-90759-6)
Supplement: Supplementary file 1 — Supplementary Information. [file 41598_2021_90759_MOESM1_ESM.pdf]

Supplementary Information for

Trace compounds in Early Medieval Egyptian blue carry information on provenance, manufacture, application, and ageing

Petra Dariz[1,ǂ], and Thomas Schmid[1,2,ǂ,*]

[1] Humboldt-Universität zu Berlin, School of Analytical Sciences Adlershof (SALSA), 10099 Berlin, Germany

[2] Bundesanstalt für Materialforschung und -prüfung (BAM), 12489 Berlin, Germany

[*]thomas.schmid@bam.de or thomas@schmid.eu.com

[ǂ]these authors contributed equally to this work

Raman and luminescence spectra of cuprorivaite and carbon black

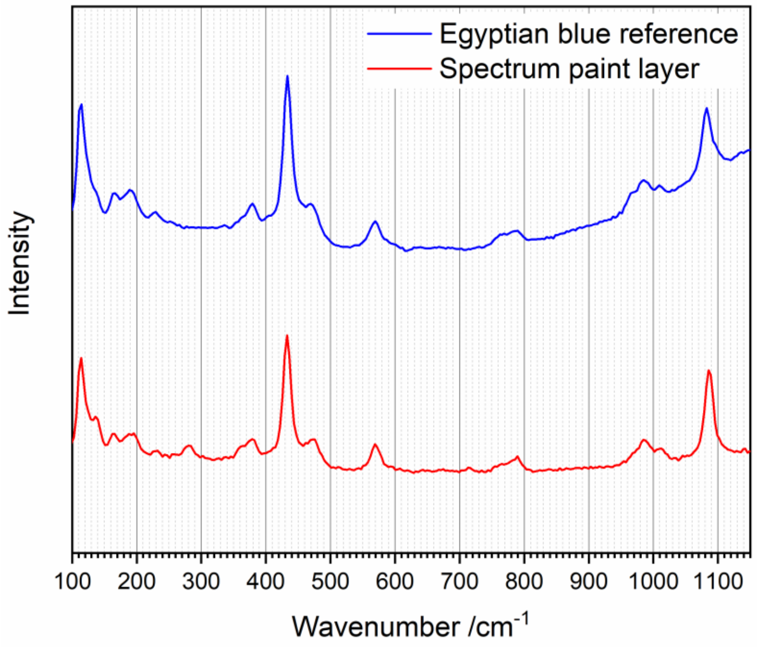

Figure S1. Raman spectra of cuprorivaite in the paint layer (red) and of commercially available Egyptian blue employed as reference (blue).

Egyptian blue was proven as the pigment of the monochrome paint layer under study by comparing sample spectra with spectroscopic data of modern Egyptian blue from Kremer Pigmente GmbH & Co. KG (Aichstetten, Germany), which were acquired with the same instrument. The good agreement of the wavenumber positions of Raman bands in both spectra proofs the presence of cuprorivaite in the sample material (see Fig. S1). According to the literature, all Raman bands in the reference spectrum can be assigned to cuprorivaite (the sample spectrum might by superimposed with weak contributions from other minerals), including signals at 1013 cm^{-1} and 1082 cm^{-1}, which some authors interpreted as bands from sulphates and carbonates, respectively, due to their positions coincidentally matching according typical spectral ranges [52]. Differences in terms of relative Raman band intensities and – as a consequence – missing weak peaks in either the sample or reference spectrum can be explained by polarisation effects due to different crystal orientations (see, for example, Ref. [44] and further explanations below). In addition to the characteristic Raman bands, the well-known luminescence of cuprorivaite was detected in the red to near-infrared spectral region with its maximum at around 900 nm (see Fig. S2) [28, 49-54].

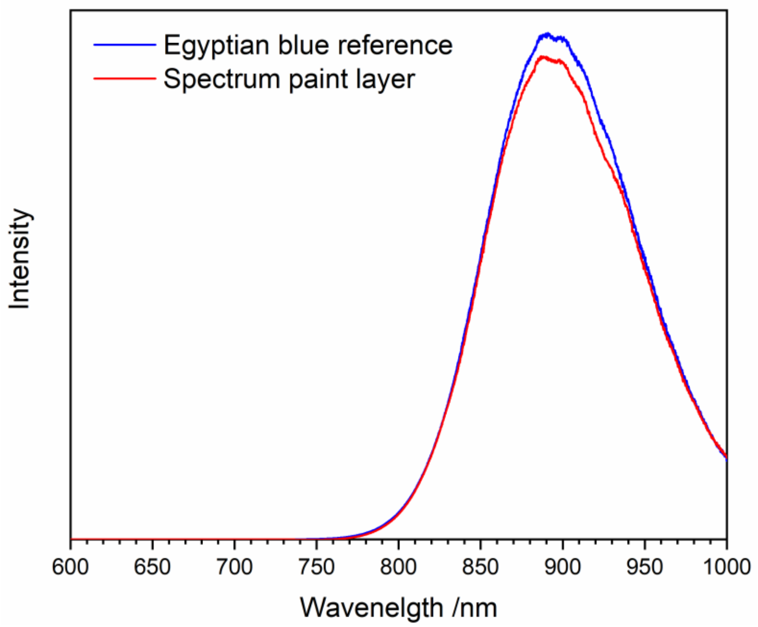

Figure S2: Luminescence emission of cuprorivaite excited in the paint layer (red) and a commercially available Egyptian blue employed as reference (blue) by a 532-nm laser.

Also the luminescence spectra reveal good agreement between sample and reference data. The spectra shown in Fig. S2 were acquired with strongly reduced laser power and acquisition time, i.e. 0.01% or 4 µW, respectively, and 0.1 s per acquisition (compare Methods section in the main text) and intensity-corrected by using the instrument control software Labspec 6 (Horiba JobinYvon) in order to compensate for the strong decrease of charge coupled device (CCD) detector sensitivity towards high wavelengths. Because of almost complete sensitivity loss at 1000 nm, the spectrum is cut at that wavelength. The intensity correction shifted the seeming emission maximum from 877 nm to approx. 895 nm, and its deviation from the generally accepted value of 910 nm is most likely due to incomplete sensitivity compensation, while shape and fine structure of the broad luminescence band are in good agreement with published data [49, 50].

In addition to the red to near-infrared luminescence of cuprorivaite, an orange to red emission (maximum around 630 nm) was detected in the paint layer and assigned to carbon black from the underpainting due to its co-localisation with the Raman signature of amorphous carbon consisting of broad and overlapping D and G bands at approx. 1375 cm^{-1} and 1600 cm^{-1}, respectively (see Fig. S3) [S1].

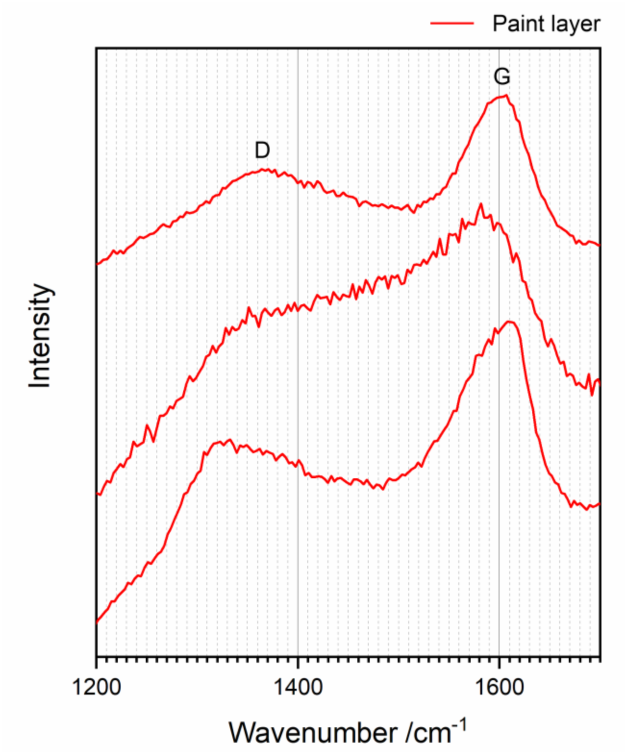

Figure S3: Examples of Raman spectra of amorphous carbon detected in the paint layer due to the presence of carbon black in the underpainting revealing the characteristic G and D bands. Most carbon spectra of the paint layer are best described by the shape of the labelled spectrum shown on the top.

Fig. S4 reveals examples of luminescence spectra taken from the same Raman map of the paint layer including spectra mainly assigned to amorphous carbon (including Raman D and G bands) or cuprorivaite with some luminescence emission intensities reaching the saturation level of the CCD detector at approx. 65,000 counts. Fig. S5 shows the Raman map with the distributions of the Raman G band intensity of amorphous carbon and the fluorescence emission intensity evaluated at 600 nm, slightly off the estimated maximum in order to reduce crosstalk with the emission of cuprorivaite.

The observations described here and schematic spectra shown in Ref. [52] suggest that Raman microspectroscopy can measure both, Raman and luminescence spectra of cuprorivaite simultaneously. Please, consider the weakness of the Raman spectrum with a maximum of approx. 200 counts above the background level (inset in Fig. S4) in comparison to the luminescence in the shown spectral range (almost) hitting the saturation level at approx. 65,000 counts. When the full luminescence spectrum in Fig. S2 is taken into account, the high difference in quantum efficiencies of Raman scattering and luminescence emission becomes obvious, as the emission in the limited spectral range shown in Fig. S4 is hidden below the noise, if instrument settings are optimised to keep the emission at the maximum (at around 900 nm) slightly below the saturation level.

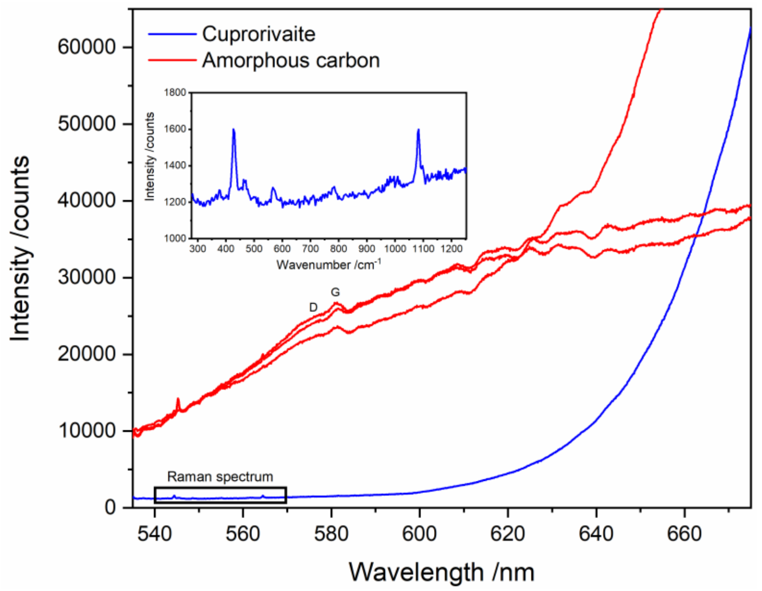

Figure S4: Luminescence spectra detected at different spots of the paint layer, which can be mainly assigned to amorphous carbon (red) or cuprorivaite (blue).

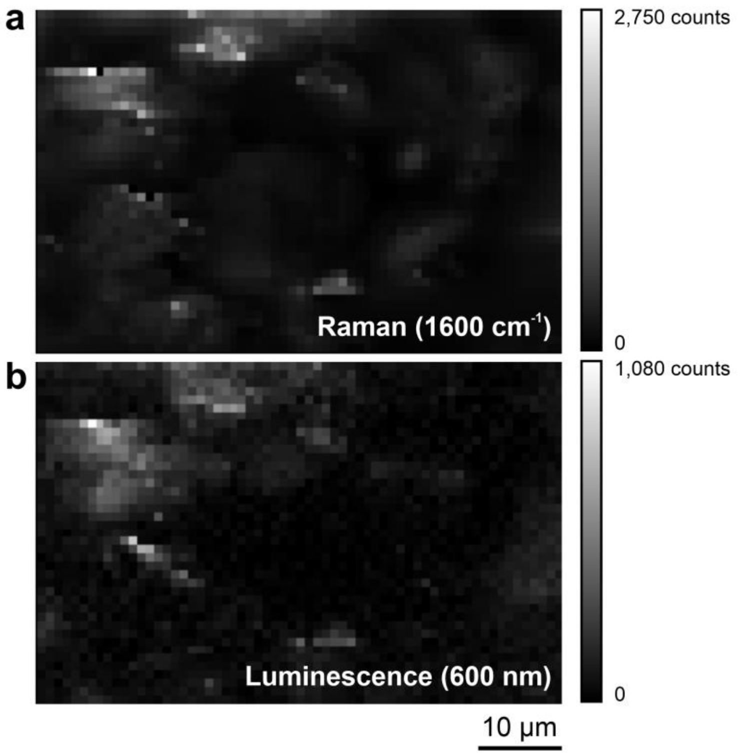

Figure S5: Distribution maps of the intensities of the Raman G band of amorphous carbon at 1600 cm^{-1} and a spectrally broad, orange to red luminescence emission evaluated at 600 nm. Co-localisation of both effects proofs the assignment of this luminescence to the carbon black of the underpainting.

Based on the differences in acquisition times and laser powers (factor of 1,000 in laser power and factor of 10 in acquisition times) and two orders of magnitude difference in signal intensities if the luminescence is evaluated far from its maximum (see Fig. S4), increasing to more than six orders of magnitude at the emission maximum (see Fig. S2), we conservatively estimate a factor of at least six orders of magnitude between luminescence emission and Raman scattering. Thus, due to the limited dynamic range of detectors, the full Raman and luminescence spectra cannot be detected simultaneously, and also the experiments described in Ref. [52] were performed with two consecutive measurements having accordingly optimised parameters. Previous studies have shown that only in some cases of resonance Raman scattering, Raman bands can be barely observed, if fluorescence emission is tuned to slightly below the saturation level, but even then, two subsequent measurements with adapted parameters must be performed in order to achieve Raman spectra of reasonable quality [S2]. A compromise is to evaluate the strong cuprorivaite luminescence emission far from its maximum. In the present case, we evaluated the carbon fluorescence at 600 nm and the luminescence emitted by cuprorivaite at 640 nm. That way, with the measurement parameters listed in the Methods section in the main text, saturation of the luminescence signal was avoided, while acquiring Raman spectra of reasonable quality.

According to Fig. S4, the luminescence emission evaluated at 640 nm exhibits some crosstalk with the carbon fluorescence. Therefore, some of the carbon structures are seen in the luminescence image in Fig. S6a (for example, in the top left corner). In order to improve the contrast between the emissions of cuprorivaite and carbon, the ratios of the signals at 640 nm and 600 nm were calculated and their distribution plotted in Fig. S6b. The most prominent Raman band of cuprovivaite at 430 cm^{-1} reveals the same distribution pattern (Fig. 6c). The same applies to the cuprorivaite band at 981 cm^{-1}, only with the difference of a strongly reduced signal-to-noise ratio due to the relative weakness of this band, and the missing of some of the cuprorivaite crystals in this spectroscopic image as a consequence. Thus, we cannot confirm the correlation of the Raman band at 981 cm^{-1} with luminescing cuprorivaite along with the presence of a non-luminescing silicate species in the mixture revealing the same Raman spectrum except this band, as hypothesised in Ref. [52]. We rather follow the interpretation by Ariadne Kostomitsopoulou Marketou et al. [44] that all Raman bands seen in Fig. S1 can be assigned to cuprorivaite, and their relative intensities only change due to different crystal orientations (compare our previous studies on qualitative [35] and quantitative uses [45] of polarisation effects for Raman imaging).

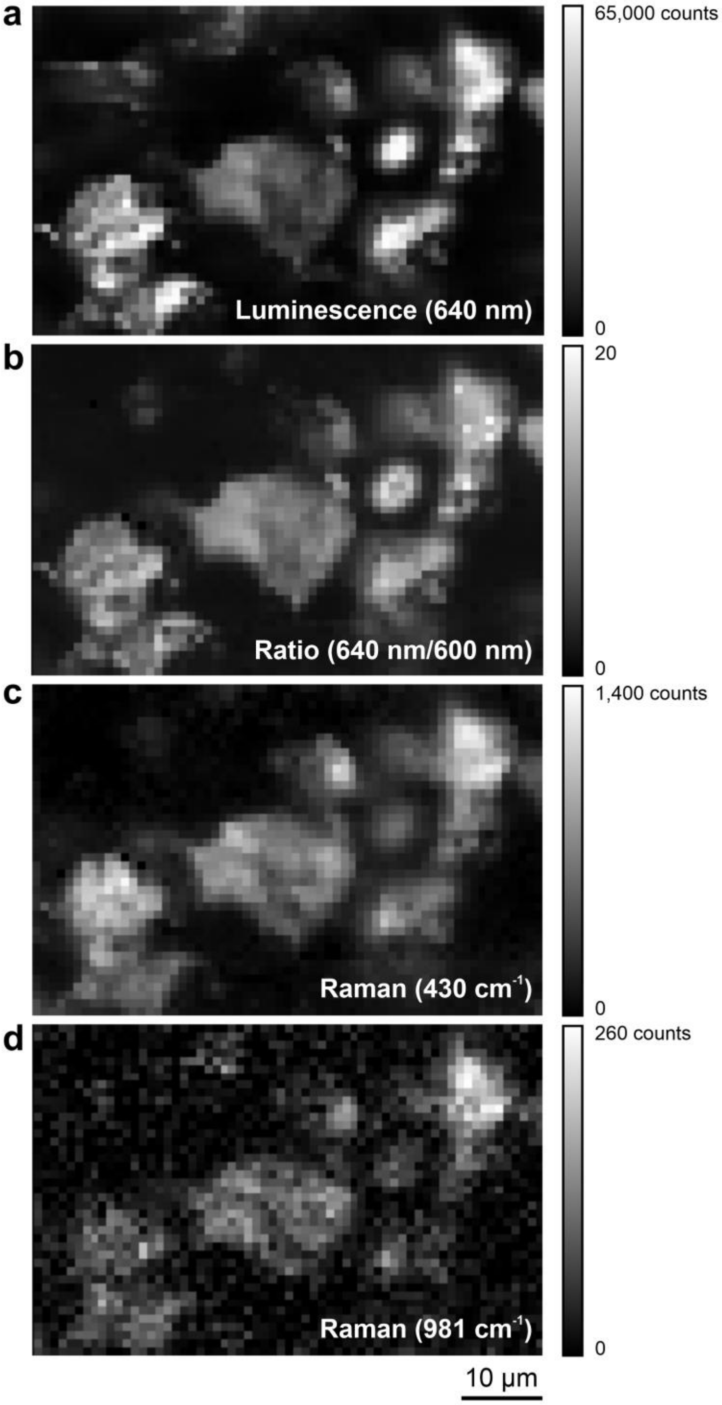

Figure S6: Distribution maps of (a) the luminescence emission intensity at 640 nm, (b) the ratio of luminescence emissions at 640 nm and 600 nm, and the Raman intensities at (c) 430 cm^{-1} and (d) 981 cm^{-1} acquired within the paint layer.

Assignments of Raman spectra of the Egyptian blue paint layer

Examples of spectra from the Egyptian blue paint layer and according reference data are presented in the following sections (see Figs. S7 and S9–S20). All spectra are shown as measured, except for sample spectra marked with an asterisk (*), which were baseline-corrected for better comparability. Trace components are grouped according to chemical aspects, for example into carbonates and sulphates. The groups are arranged according to the position of their lead element within the periodic table, from carbonates (C, group IVA) to iron oxides (Fe, group VIIIB). The spectra collection ends with oxalates and further organic compounds.

Main components: Cuprorivaite, quartz, calcite, and feldspars

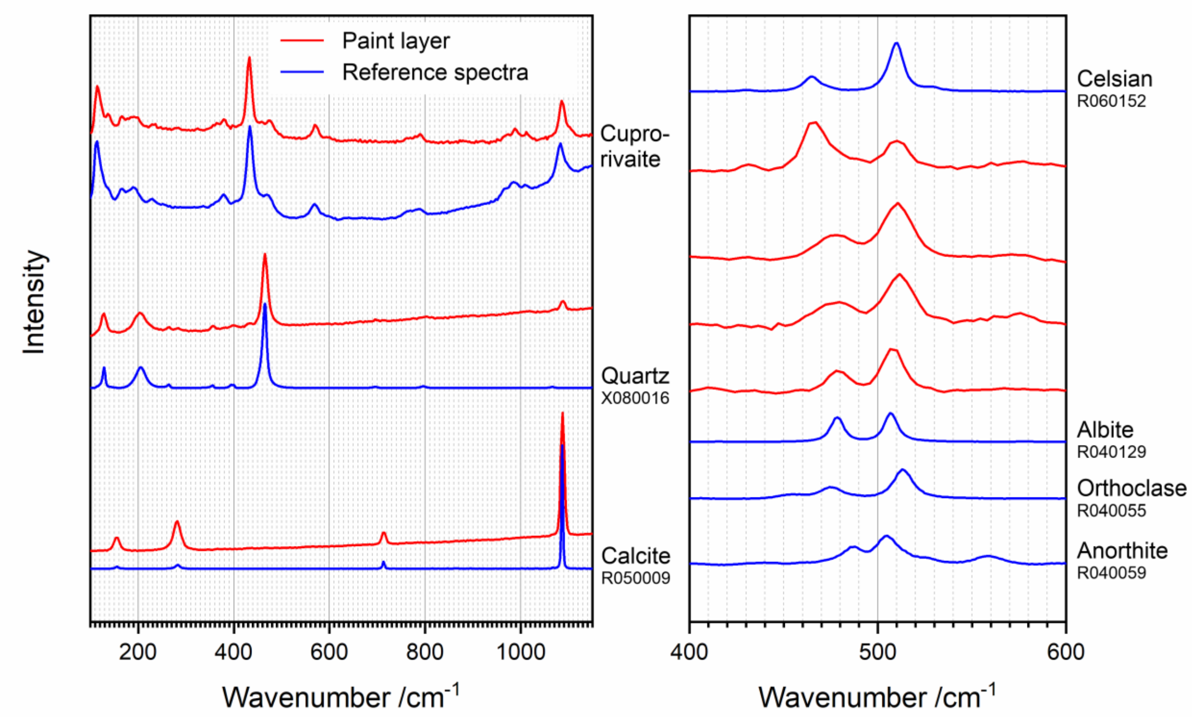

Figure S7: Raman spectra of the paint layer (red) assigned to main components of Egyptian blue by comparison with reference spectra (blue).

Raman spectra were assigned to mineral phases by comparison with either the spectrum of a commercially available cuprorivaite (see above) or with spectra from the rruff spectral library [47]. In the latter case, rruff ID numbers of the format Xnnnnnn or Rnnnnnn (with n = 0…9) are given in the figures. If according reference spectra were not available in the rruff database, sample spectra were compared with literature data (see examples in the following sections). As revealed by Fig. S7, spectra were assigned based on matching wavenumbers of Raman bands. Relative band intensities might vary in some cases due to crystal orientation effects. Also band widths can differ between sample and reference data either due to different crystallinities or simply because of the typically higher spectral resolution of the reference data; in order to achieve reasonable measurement times of the 166,477 spectra measured during this study in total, a spectral resolution of approx. 3 cm^{-1} was applied enabling the acquisition of the full spectral range of 50 to 3200 cm^{-1} within a single acquisition.

Individual members of the feldspars $M(I)_xM(II)_{1-x}Al_{2-x}Si_{2+x}O_8$ and their solid solution series can be identified by determining exact band positions [S3]. The four red examples of feldspar spectra from the paint layer shown in Fig. S7 can, thus, be either assigned to celsian or albite, or – in case of intermediate band positions – to members of the solid solution series albite–orthoclase. By conducting Lorentzian peak fitting in each pixel that was assigned to feldspars, this effect was used to image local compositional variations within this alkali feldspar series (see Fig. S8).

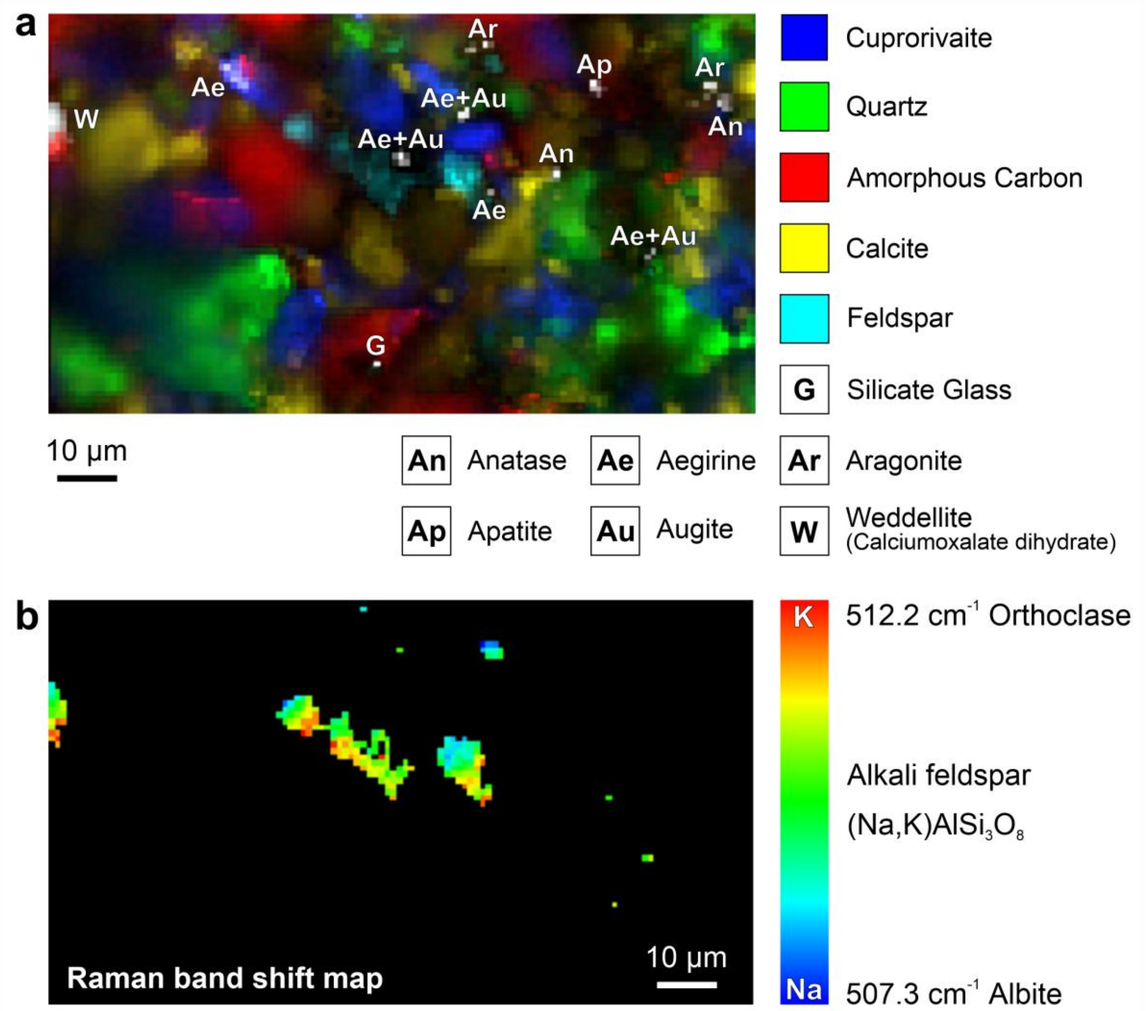

Figure S8: (**a**) Raman phase distribution map of the paint layer surface based on the intensities of marker bands, and (**b**) chemical composition map of the feldspars detected within this area based on the determination of exact Raman shifts by Lorentzian peak fitting.

Carbonates: Calcite, aragonite, dolomite

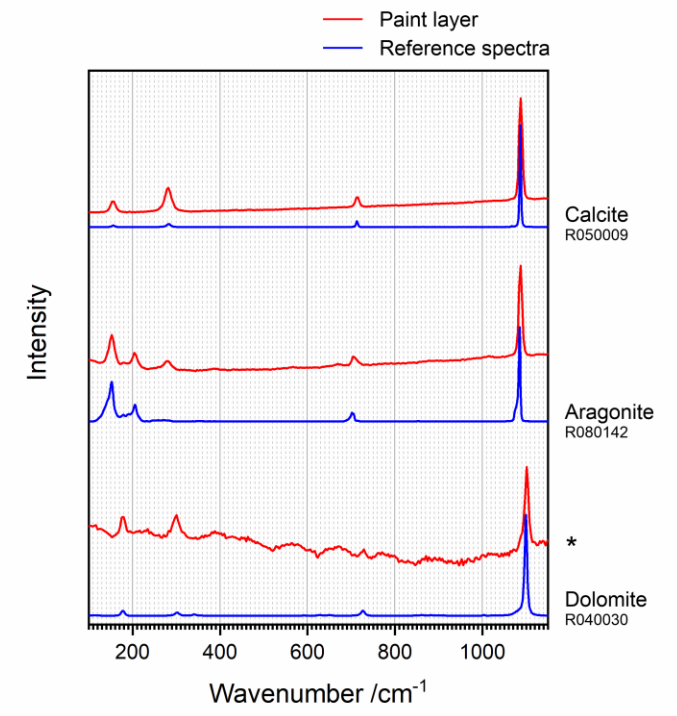

Figure S9: Raman spectra of the paint layer (red) assigned to carbonates by comparison with reference spectra (blue). The spectrum marked with an asterisk (*) was baseline-corrected by subtracting a linear function.

The polymorphic calcium carbonates $CaCO_3$ calcite and aragonite can theoretically be discriminated based on the positions of their most prominent Raman band assigned to the symmetric carbonate stretch vibration at 1086 cm^{-1} for calcite and 1085 cm^{-1} for aragonite, a difference not accessible with the limited spectral resolution of approx. 3 cm^{-1} applied in this study. Assignment of aragonite spectra was feasible by evaluating a broadened band appearing at 701 cm^{-1} (consisting of a doublet only visible at higher spectral resolutions, compare Ref. [S4]), whose counterpart in the calcite spectrum is at 713 cm^{-1} (see Fig. S9). This assignment is confirmed by the characteristic spectral variations in the low-wavenumber region below 300 cm^{-1}. The calcium magnesium carbonate dolomite $CaMgCO_3$ reveals its symmetric carbonate stretch vibration at a significantly different Raman band position of 1098 cm^{-1}.

Silicon and lead oxides: Quartz, cristobalite, silicate glass, and lead oxide

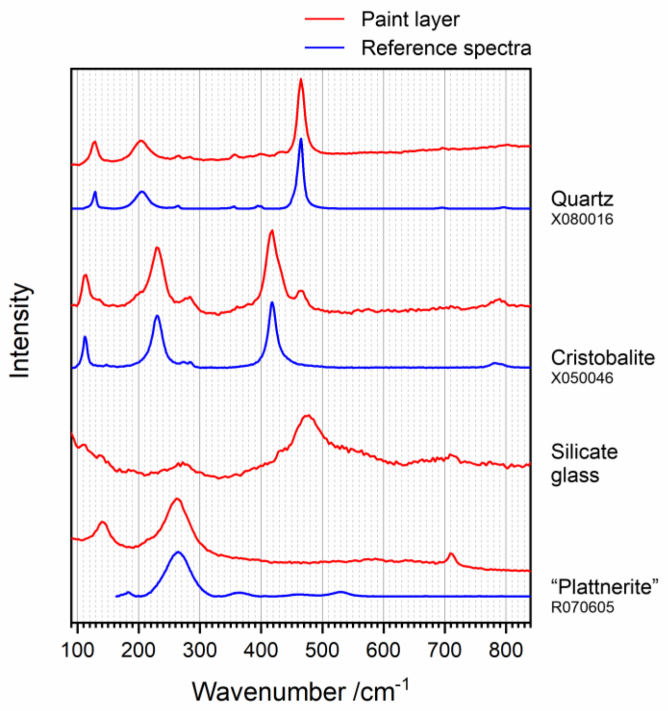

Figure S10: Raman spectra of the paint layer (red) assigned to silicon oxides SiO_2 and lead oxides PbO_x by comparison with either reference spectra (blue) or literature data (see text for explanation).

The spectrum of silicate glass (see Fig. S10) was assigned by comparison with literature data. Most characteristic of the spectrum is a Raman band at approx. 475 cm^{-1} that is broadened (due to the amorphous character) and shifted with respect to the most prominent band of quartz at 465 cm^{-1}. Raman spectra of volcanic glasses with high SiO_2 and accordingly low iron content include the same asymmetric most prominent peak [85-87].

The exact stoichiometry of the lead oxide compounds in the paint layer cannot be determined by Raman spectroscopy, as plattnerite PbO_2 is known to easily decompose into massicot PbO because of the action of the laser employed in a Raman experiment [84]. Hence, the detected spectrum can be assigned to massicot when taking into account shifts of Raman bands due to heating. Spectrum R070605 assigned to plattnerite in the rruff spectral library in reality represents the signature of heated massicot and is in good agreement with our observation. Literature spectra presented in Ref. [84] include both most prominent bands appearing at approx. 138 cm^{-1} and 262 cm^{-1} in the sample spectrum (the rruff spectrum was acquired within a restricted range and misses the low-wavenumber band).

Pyroxenes (inosilicates): Augite, aegirine, and diopside

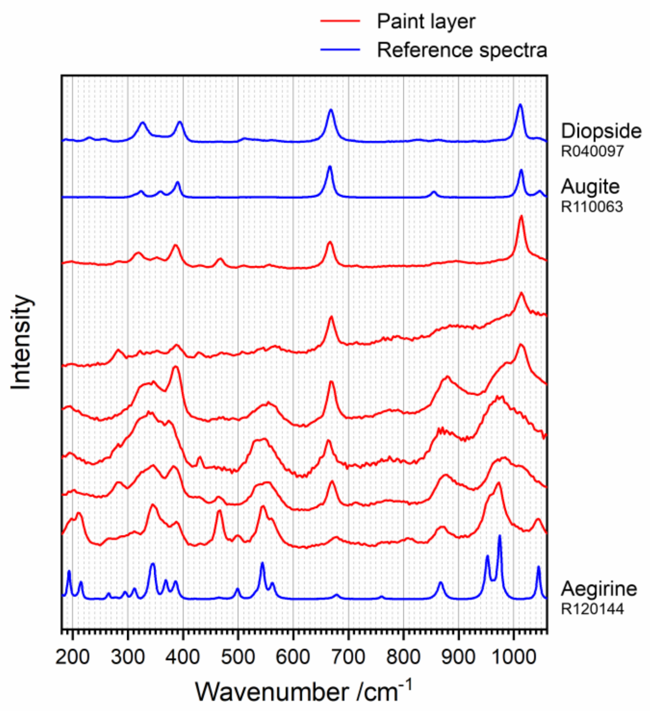

Figure S11: Raman spectra of the paint layer (red) assigned to the pyroxenes augite $(Ca,Mg,Fe)_2Si_2O_6$ and aegirine $NaFeSi_2O_6$ by comparison with reference spectra (blue), which do not allow the discrimination between augite and diopside. Therefore, these two phases are subsumed under 'augite' throughout this study. The sample spectra (red) can be assigned to aegirine, augite and mixtures of both.

Tin oxides and silicates: Cassiterite and malayaite

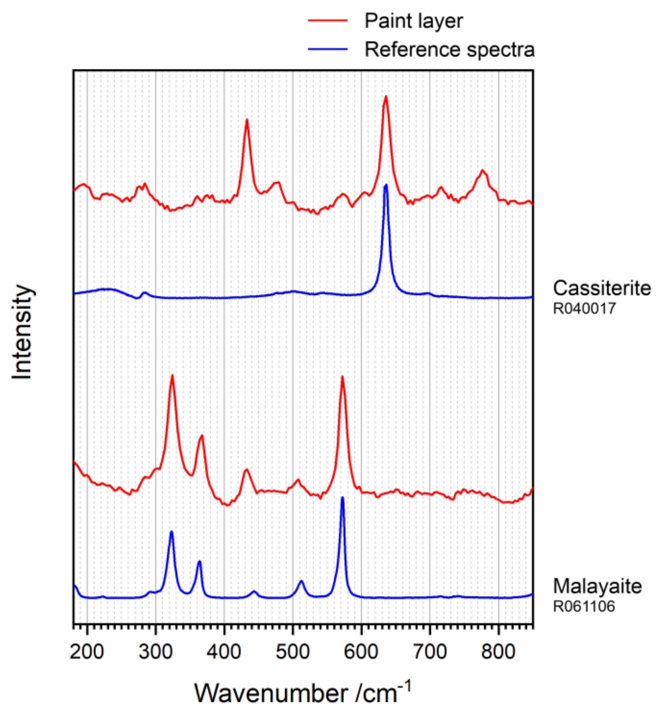

Figure S12: Raman spectra of the paint layer (red) assigned to cassiterite SnO_2 (here, superimposed with characteristic bands of cuprorivaite) and malayaite $CaSnOSiO_4$ by comparison with reference spectra (blue).

Phosphates and arsenates: Apatite and basic copper arsenate

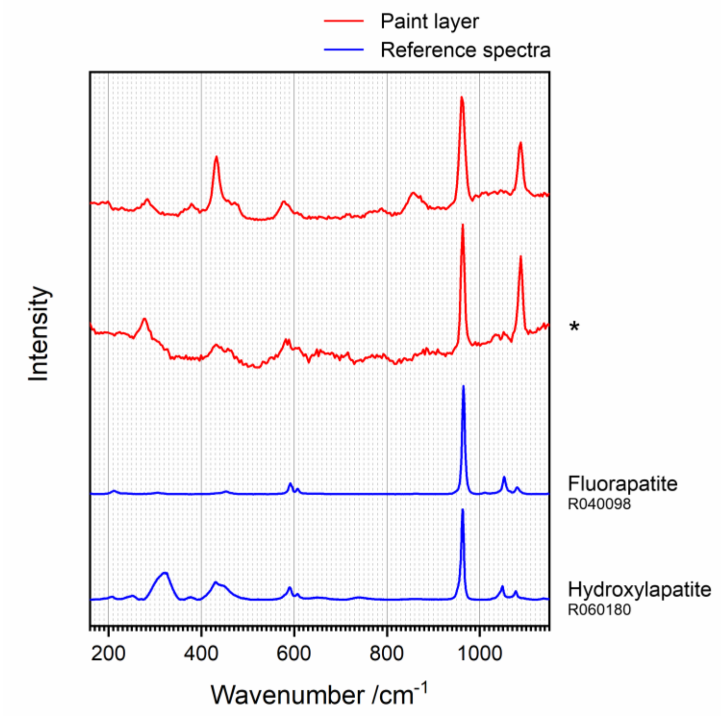

Figure S13: Raman spectra of the paint layer (red) assigned to apatite compared with reference spectra of fluorapatite and hydroxylapatite (blue). The spectrum marked with an asterisk (*) was baseline-corrected by subtracting a linear function.

Apatite $Ca_5(PO_4)_3(F,OH)$ in the complex mixture of the Egyptian blue paint layer could be identified due to its strong symmetric phosphate stretch mode at 962–963 cm^{-1}, but because of overlap of the much less intense further Raman bands with the signatures of other phases and the high similarities between reference spectra available so far, further distinguishment into fluorapatite, hydroxylapatite, or according solid solution series was not possible (see Fig. S13).

Similarly, sample spectra of arsenates could be assigned to mineral phases with only limited precision. Most intense and characteristic part of the spectrum is a broad and asymmetric peak at around 840 cm^{-1}, which appears at slightly shifted positions in the spectra of different individual crystals in the paint layer and can be deconvoluted into three broad and overlapping bands at, for example, 781 cm^{-1}, 837 cm^{-1}, and 884 cm^{-1} (see Fig. S14). These spectra best agree with reference spectra of the dimorphic basic copper arsenates clinoclase/gilmarite $Cu_3(AsO_4)(OH)_3$ und cornubite/cornwallite $Cu_5(AsO_4)_2(OH)_4$. While a comparison with rruff spectra seems to speak for clinoclase (a reference spectrum of cornubite is not (yet) available at rruff), a look into the literature reveals a more complex picture [77-79]. Therefore, we assign this type of spectra to basic copper arsenate with the general formula $Cu_x(AsO_4)_y(OH)_{2x-3y}$.

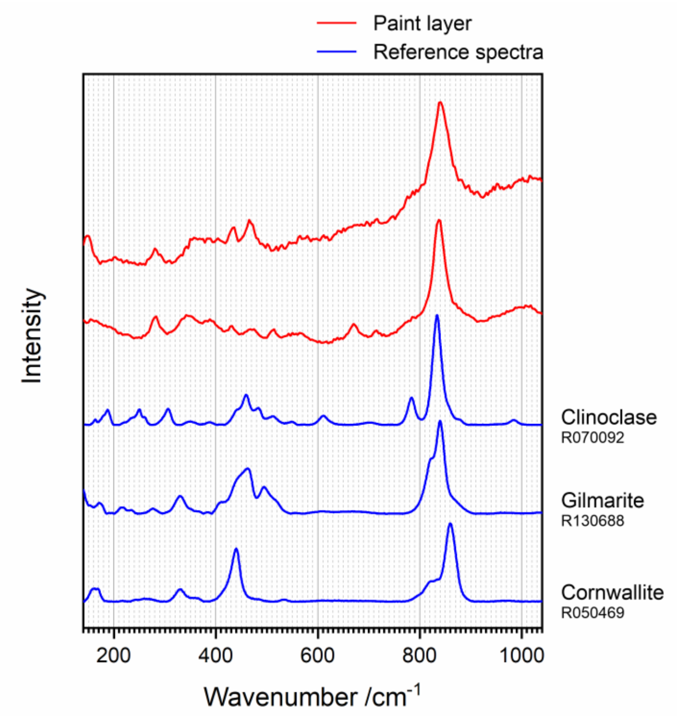

Figure S14: Raman spectra of the paint layer (red) assigned to basic copper arsenate compared with reference spectra of clinoclase, gilmarite, and cornwallite (blue).

Sulphates: Syngenite, polyhalite, osarizawaite, and natrojarosite

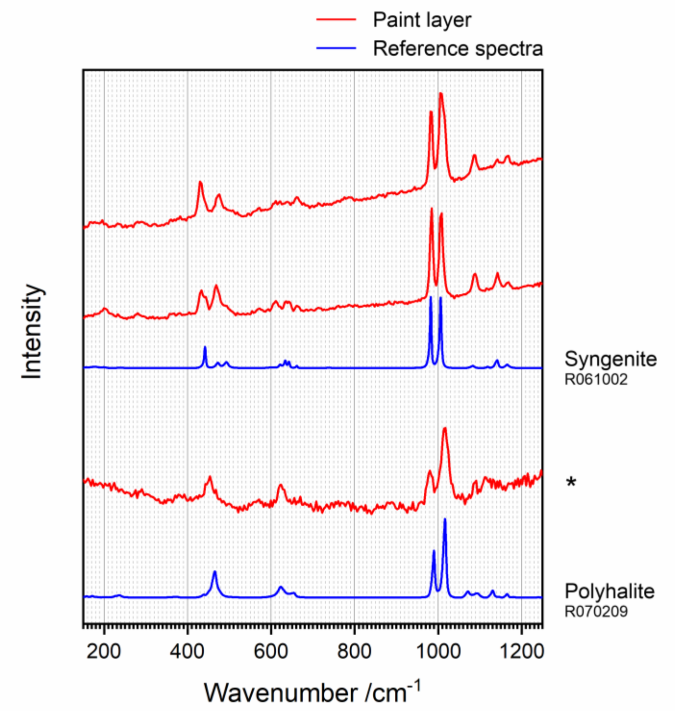

Figure S15: Raman spectra of the paint layer (red) assigned to the hydrated sulphates syngenite and polyhalite by comparison with reference spectra (blue). The spectrum marked with an asterisk (*) was baseline-corrected by subtracting a linear function.

The hydrated sulphates syngenite $K_2Ca(SO_4)_2 \cdot H_2O$ and polyhalite $K_2Ca_2Mg(SO_4)_4 \cdot 2H_2O$ can be identified based on their characteristic doublets at 982 cm^{-1}/1006 cm^{-1} and 980–990 cm^{-1}/1017 cm^{-1}, respectively (see Fig. S15). The assignments are confirmed by several weak Raman bands, which were detected in many cases of occurrence of these spectra in the paint layer. A shoulder towards higher wavenumbers of the 1006-cm^{-1} band in syngenite spectra (as, for example, in the top spectrum in Fig. S15) is interpreted as local mixture with polyhalite.

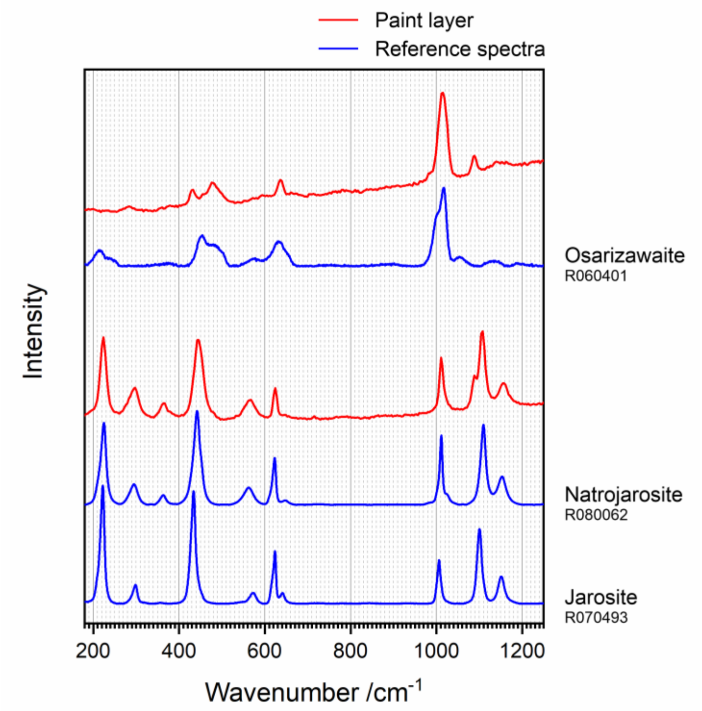

Figure S16: Raman spectra of the paint layer (red) assigned to the hydrated hydroxyl sulphates osarizawaite and natrojarosite by comparison with reference spectra (blue).

A characteristically broadened signal peaking at approx. 1015–1016 cm^{-1} consisting of two or three overlapping bands was assigned to osarizawaite $PbCuAl_2(SO_4)_2(OH)_6$ (see Fig. S16). The spectrum of the other hydrated hydroxyl sulphate natrojarosite $NaFe_3(SO_4)_2(OH)_6$ contains several strong bands, for example, at 1011 cm^{-1} and 1110 cm^{-1}, and is significantly different from the signature of the potassium analogue jarosite $KFe_3(SO_4)_2(OH)_6$ with according peaks at 1006 cm^{-1} and 1100 cm^{-1}. The latter was not detected within the paint layer.

Copper sulphides and oxides: Chalcocite and copper oxide

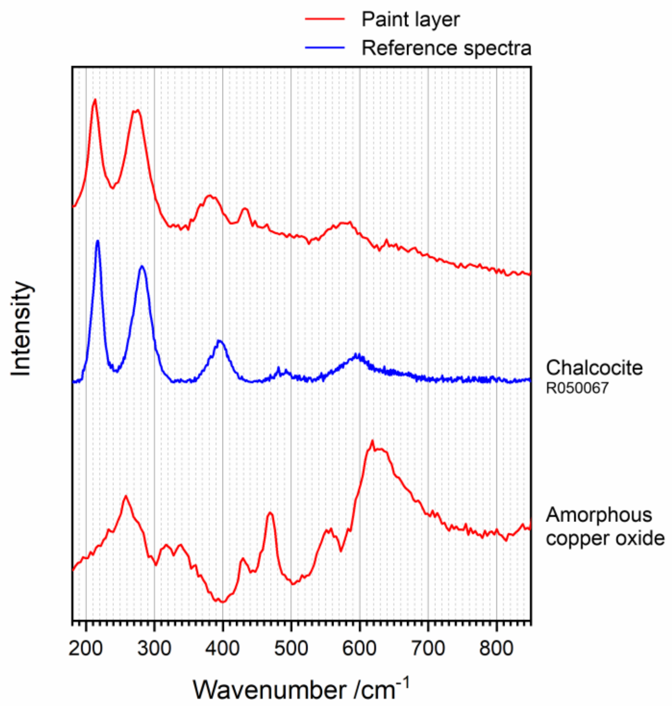

Figure S17: Raman spectra of the paint layer (red) assigned to chalcocite and amorphous copper oxide by comparison with reference spectra (blue) or literature data (see text for explanation).

Chalcocite Cu_2S can be identified based on its broad most prominent Raman bands at 214–218 cm^{-1} and 270–280 cm^{-1} (see Fig. S17). When detecting this spectrum, the presence of chalcopyrite $CuFeS_2$ cannot be excluded, because of the potential conversion of this mineral phase into chalcocite due to heating by the laser during a Raman experiment [72].

Indicators for copper oxides within the paint layer were not the Raman spectra of the crystalline phases tenorite CuO and cuprite Cu_2O, but Raman signatures consisting of several broad bands were detected as shown in Fig. S17, typically overlapped with sharp peaks from, for example, cuprorivaite and quartz. Such spectra, appearing in the paint layer with slight variations in peak widths and positions, are in good agreement with literature data assigned to amorphous (or nanocrystalline) copper oxides of undefined stoichiometry Cu_xO [73].

Titanium and chromium oxides: Anatase and eskolaite

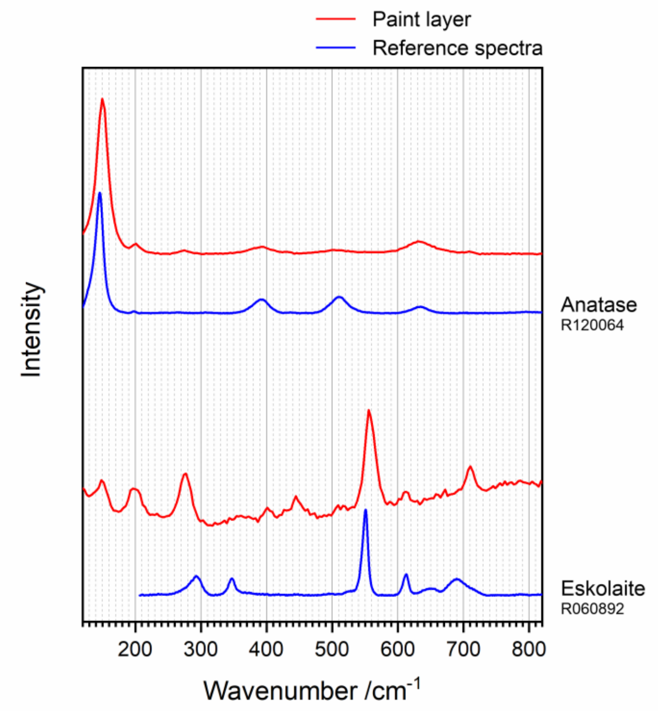

Figure S18: Raman spectra of the paint layer (red) assigned to anatase and eskolaite by comparison with reference spectra (blue).

The TiO_2 polymorph anatase can be reliably identified due to its very strong most prominent Raman mode appearing at positions varying approximately between 145 cm^{-1} and 150 cm^{-1} (see Fig. S18). Raman band shifts and widths of anatase are known to vary in nanoparticles due to quantum confinement. In the spectra of particles with ≤ 28 nm diameter, the frequencies of Raman modes and their band widths increase as a function of decreasing size [67]. The most prominent E_g mode in some Raman spectra of the paint layer coincides with the according peak in the reference spectrum (RRUFF R120064) at 146 cm^{-1} (12 cm^{-1} full width at half maximum, FWHM) corresponding to a diameter of ≥ 28 nm, while others appear shifted to ~ 150 cm^{-1} (FWHM ≈ 22 cm^{-1}) and thus, indicate nanometric small anatase (according to the data in Ref. [67] approx. 8 nm diameter).

In case of eskolaite Cr_2O_3, typically both most prominent bands appearing at 555–559 cm^{-1} and 612 cm^{-1} could be detected within the paint layer even if the spectrum was superimposed by contributions from other mineral phases (like calcite in the sample spectrum shown in Fig. S18). Co-localisation of these bands in Raman maps (data not shown) confirms the assignment of both bands to the same compound.

Iron oxides: Magnetite, jacobsite, and hematite

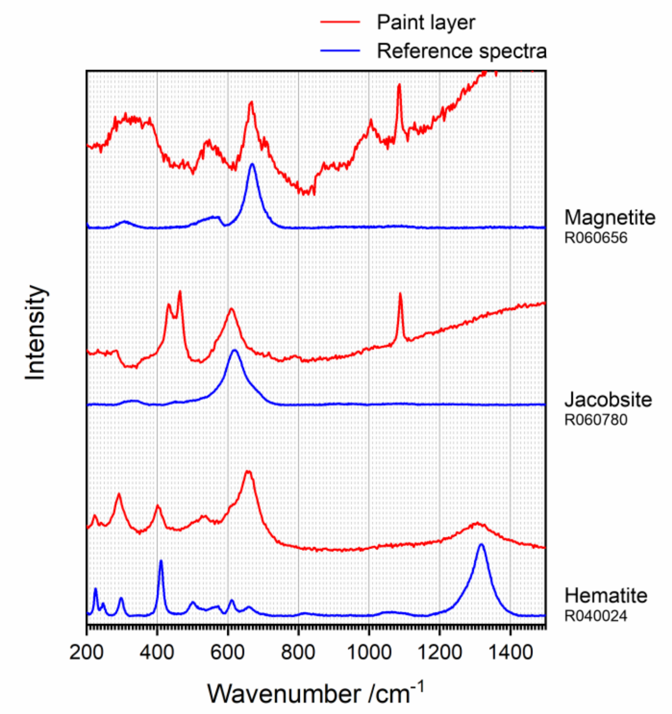

Figure S19: Raman spectra of the paint layer (red) assigned to magnetite, jacobsite, or hematite by comparison with reference spectra (blue).

The Raman spectra of the spinels magnetite Fe_3O_4 and jacobsite $MnFe_2O_4$ are mainly characterised by broad bands appearing at two shifted positions: 665–669 cm^{-1} (Fe_3O_4) and 610–618 cm^{-1} ($MnFe_2O_4$). Presence of magnetite confirms its successful protection from a potential conversion into hematite by laser heating [46]. As explained in the Methods section in the main text, the laser power was reduced from 40 mW to 10 mW (at the sample surface) for this reason. Differences between sample spectra of hematite α-Fe_2O_3 and according reference data are known to occur due to integration of foreign ions into the crystal lattice; Fe^{3+} can be easily exchanged with, for example, Al^{3+} due to same charges and similar ionic radii [S5-S8]. Changes of the masses and force constants induce band shifts, and the symmetry break due to different ions enables the detection of modes that are Raman-inactive in pure hematite. Most prominent example of the latter effect is the raise of a broad, Raman-forbidden mode at approx. 655–660 cm^{-1}, very similar to the most prominent band of magnetite. For this reason, the identification of hematite was confirmed in every measurement by a visual check for the presence of the low-wavenumber modes appearing at 225 cm^{-1}, 296 cm^{-1}, and 411 cm^{-1} in the reference spectrum and at accordingly shifted positions in the sample data. In cases of low autofluorescence background, also the second order mode of hematite at approx. 1318 cm^{-1} (or shifted to higher wavenumbers in case of Al^{3+} integration) can be detected (compare the evaluation of Raman band shifts for imaging compositional variations in hematite presented in the supplementary material of Ref. [S9]).

Oxalates: Weddellite and unknown organic salts

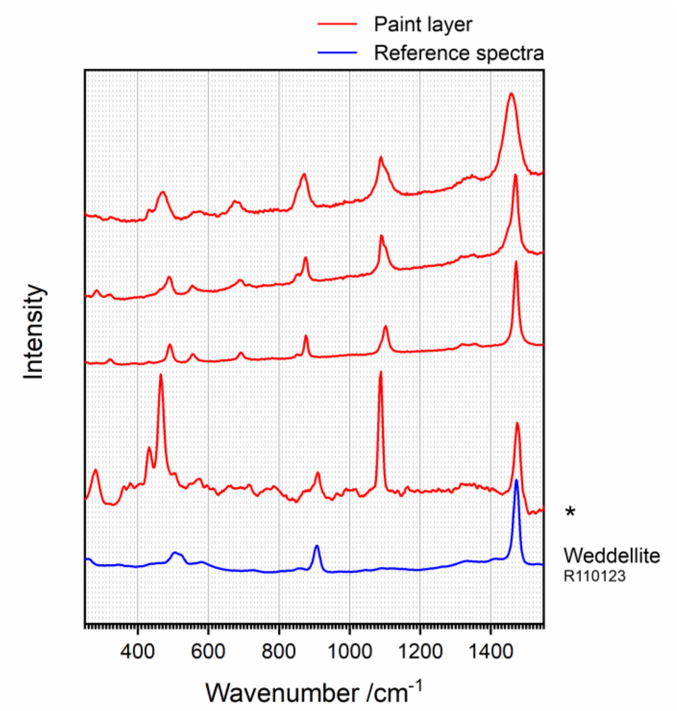

Figure S20: Raman spectra of the paint layer (red) assigned to the oxalate weddellite and further (so far unknown) organic compounds by comparison with reference spectra (blue). The spectrum marked with an asterisk (*) was baseline-corrected by subtracting a linear function.

Raman bands in the range around 1450 cm^{-1} are good indicators for the presence of organic compounds, as they typically represent C–H bending modes, which along with the according stretching modes around approx. 2900 cm^{-1} are present in the spectra of all hydrocarbons. The only potential candidates for these modes detected in the paint layer were bands in the range of 1459 cm^{-1} to 1475 cm^{-1} (see Fig. S20). Comparison with literature spectra revealed them as carboxylate stretching modes of oxalates (or related compounds), while other organic compounds inducing C–H bending modes within this range were not detected, which was confirmed by the missing of C–H stretching bands. Most of these spectra were assigned to the calcium oxalate dihydrate weddellite $Ca(C_2O_4)\cdot 2H_2O$ with its most prominent mode at 1475 cm^{-1} (in Fig. S20 superimposed with contributions from cuprorivaite and quartz). The rruff data shown in Fig. S20 for comparison is the broad-scan spectrum that was baseline-corrected by subtraction of a linear function, because the normal database spectrum was cut at below 1400 cm^{-1}. The Raman spectra of the calcium oxalate monohydrate whewellite and of the oxalates of magnesium, copper, iron, sodium and ammonium are significantly different, and their presence can therefore be excluded [S10, S11]. One oxalate spot on the paint layer additionally contained organic compounds with slightly deviating spectra (see the three top sample spectra in Fig. S20) and shifts of the main band down to 1459 cm^{-1} accompanied by band broadening. Along with the second strongest mode at 872–876 cm^{-1} (907–909 cm^{-1} in the spectrum of weddellite) this does not fit any of the oxalate spectra published so far and is thus, assigned to either an oxalate with a Raman spectrum that has not been published so far or a salt of another short-chain carboxylic (di)acid.

Qualitative and quantitative evaluation of Raman maps

In each of the spectra shown above (see Figs. S7 and S9–S20) a marker band was identified, which is characteristic for one of the minerals in the paint layer. A linear baseline was subtracted around each marker band and its peak height was plotted in false colours in 2D diagrams by using own software (see Methods section in the main text) to reveal the distribution of each phase (see Figs. 4 and 5 in the main text and Fig. S8). Table S1 includes the positions of the marker bands and the ranges for baseline correction. As in this complex mixture some marker bands appear at similar wavenumbers, spectra were visually checked and manually assigned in cases of doubt.

Table S1. Phases identified in the paint layer, their parameters for data evaluation, and quantification results.

Phase	Formula	Marker band (cm^{-1})	Baseline correction from (cm^{-1})	Baseline correction to (cm^{-1})	Assigned spectra
Cuprorivaite	$CaCu^{II}Si_4O_{10}$	432	232	633	38.85%
Calcite	$CaCO_3$	1086	1037	1111	24.78%
Amorphous carbon	C	1604	1525	1673	17.60%
Quartz	SiO_2	465	408	501	11.14%
Feldspars	$M^{I}_{x}M^{II}_{1\text{-}x}Al_{2\text{-}x}Si_{2+x}O_8$	510	243	622	3.80%
Syngenite	$K_2Ca(S^{VI}O_4)_2{\cdot}H_2O$	983	953	1124	0.77%
Weddellite (Oxalate)	$Ca(C_2O_4){\cdot}2H_2O$	1475	1388	1516	0.56%
Augite–diopside	$(Ca,Mg,Fe^{II})_2Si_2O_6$	1014	953	1058	0.45%
Aegirine	$NaFe^{III}Si_2O_6$	973	942	1050	0.42%
Chalcocite	$Cu^{I}_{2}S^{-II}$	214	188	236	0.31%
Natrojarosite	$NaFe^{III}_{3}(S^{VI}O_4)_2(OH)_6$	1108	1061	1118	0.18%
Basic copper arsenate	$Cu^{II}_{x}(As^{V}O_4)_y(OH)_{2x\text{-}3y}$	837	799	868	0.17%
Cassiterite	$Sn^{IV}O_2$	635	618	660	0.13%
Magnetite	$Fe^{II}Fe^{III}_{2}O_4$	660	540	765	0.13%
Cristobalite	SiO_2	418	353	443	0.10%
Apatite	$Ca_5(P^{V}O_4)_3(F,OH)$	961	937	1056	0.10%
Osarizawaite	$Pb^{II}Cu^{II}Al_2(SO_4)_2(OH)_6$	636	615	696	0.08%
Eskolaite	$Cr^{III}_{2}O_3$	559	534	583	0.08%
Malayaite	$CaSn^{IV}OSiO_4$	324	306	342	0.08%
Polyhalite	$K_2Ca_2Mg(S^{VI}O_4)_4{\cdot}2H_2O$	1017	997	1041	0.06%
Anatase	TiO_2	150	135	161	0.06%
Copper oxide	$Cu^{I,II}_{x}O$	632	504	806	0.05%
Aragonite	$CaCO_3$	701	630	738	0.03%
Lead oxide	$Pb^{II,IV}O_x$	262	220	327	0.03%
Dolomite	$CaMg(CO_3)_2$	1098	1085	1115	0.02%
Jacobsite	$Mn^{II}Fe^{III}_{2}O_4$	610	500	730	0.01%
Hematite	$Fe^{III}_{2}O_3$	663	585	726	0.01%
Silicate glass	"SiO_2"	486	456	507	0.01%

In cases of elements with several usual oxidation numbers, these are given in the formulas as superscript Roman numerals.

For quantitative evaluation, threshold values were defined for each phase to remove pixels with low intensities in order to exclude contributions from de-focused areas in the maps of the corrugated surface of the paint layer and to avoid overestimation of the main components. For pixels assigned to a number of n components (n > 1 for co-localised minerals) a value of 1/n was added to the sum of pixels related to each of these compounds. Mainly due to the non-flat sample surface and the exclusion of low-intensity contributions, only 88,932 out of the measured 166,477 pixels were quantitatively evaluated. Table S1 lists the percentages of pixels assigned to each compound. We would like to point out that this should not be misunderstood as a quantification of a volume percentage (or even weight percentage) as it would be delivered by a bulk analysis. The quantification results in numbers of spectra (or pixels, respectively) enabling estimations of area fractions. It is intended to provide insight into the large dynamic range of this approach, enabling the determination of main components in the percent range and traces at the sub-permille level simultaneously, thus, outperforming bulk analytical techniques, such as powder X-ray diffraction. Even though for better comparability given with two decimal places, the precision of the percentages in Table S1 should not be overestimated. Nevertheless, they allow a classification of the components into percent, sub-percent and sub-permille levels (see Table 1 in the main text), enabling further interpretations of the detected minerals, for example, either as intentionally added pigments (in the percent range) or natural impurities (at the sub-percent or sub-permille levels).

Scanning electron microscopy–Energy-dispersive X-ray spectroscopy

Elemental analysis by energy-dispersive X-ray spectroscopy combined with scanning electron microscopy (SEM–EDX) averaged over an area of approx. 40 µm × 60 µm of a polished cross-section of the paint layer (see Fig. S21a) detects 13 chemical elements within this complex material: C, O, Fe, Cu, Na, Mg, Al, Si, O, S, Cl, K, and Ca, which support the assignments in Table S1 (the high intensity of the C K_α signal is due to the embedding resin). Further elements, such as Sn, Pb, Cr, and Ti were detected in measurements of other sample areas.

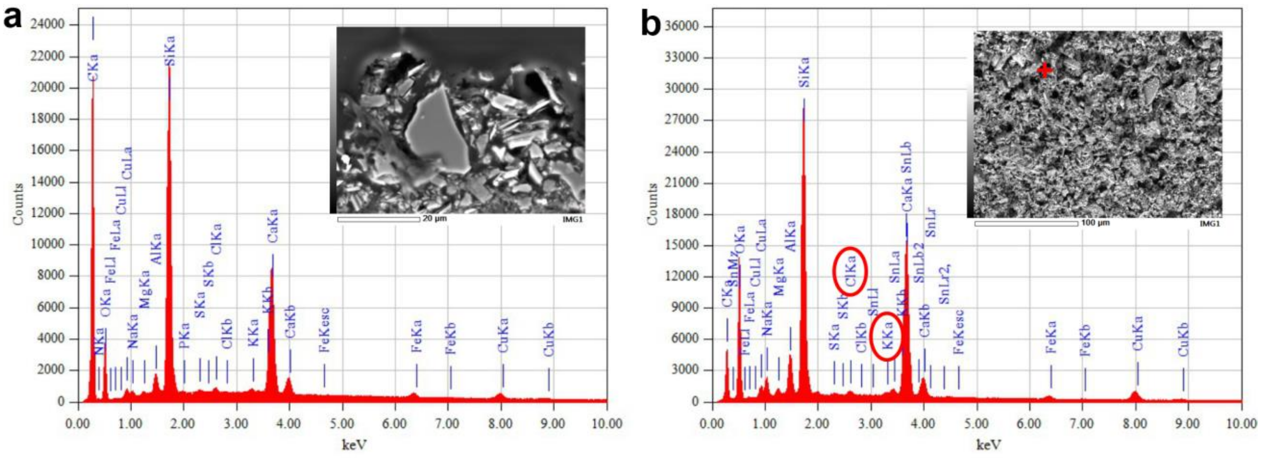

Figure S21: Energy-dispersive X-ray (EDX) spectra (**a**) averaged over the whole image shown in the inset and (**b**) performed as single-point measurement at the position marked with a red cross in the inset.

Local single-point measurements were able to detect the Raman-inactive alkali chlorides halite NaCl and sylvine KCl. Quantitative evaluation of the spectrum shown in Fig. S21b yielded a 1:1 atomic-percent ratio of K and Cl indicating a sylvine crystal within the sample (measurement performed on the surface of an uncoated patch of the pictorial layer in environmental scanning electron microscopy mode).

References

Citations in the text above of the form [x] (with x denoting a number) refer to the list of references in the main text, while literature exclusively cited within the supplementary information is designated as [Sx] and listed as follows.

S1. Bokobza, L.; Bruneel, J.-L.; Couzi, M., Raman Spectra of Carbon-Based Materials (from Graphite to Carbon Black) and of Some Silicone Composites. *C* **1**, 77-94 (2015).

S2. Urban, P. L., Schmid, T., Amantonico, A. & Zenobi, R., Multidimensional analysis of single algal cells by integrating microspectroscopy with mass spectrometry. *Anal. Chem.* **83**, 1843–1849 (2011).

S3. Freeman, J. J., Wang, A., Kuebler, K. E., Jolliff, B. L. & Haskin, L. A., Characterization of natural feldspars by Raman spectroscopy for future planetary exploration. *Can. Mineral.* **46**, 1477–1500 (2008).

S4. Dariz, P., Schmid, T., Phase composition and burning history of high-fired medieval gypsum mortars studied by Raman microspectroscopy. *Mater. Charact.* **151**, 292–301 (2019).

S5. Zoppi, A., Lofrumento, C., Castellucci, E., Dejoie, C. & Sciau, P., Micro-Raman study of aluminium-bearing hematite from the slip of Gaul *sigillata* wares. *J. Raman Spectrosc.* **37** 1131–1138 (2006).

S6. Zoppi, A., Lofrumento, C., Castellucci, E. & Sciau P., Al-for-Fe substitution in hematite: the effect of low Al concentrations in the Raman spectrum of Fe_2O_3. *J. Raman Spectrosc.* **39**, 40–46 (2008).

S7. Varnshey, D. & Yogi, A., Structural and electrical conductivity of Mn doped hematite (α-Fe_2O_3) phase. *J. Mol. Struct.* **995** 157–162 (2011).

S8. Varnshey, D. & Yogi, A., Influence of Cr and Mn substitution on the structural and spectroscopic properties of doped haematite: α-$Fe_{2-x}M_xO_3$ ($0.0 \leq x < 0.50$). *J. Mol. Struct.* **1052**, 105–111 (2013).

S9. Dariz, P. & Schmid, T., Ferruginous phases in 19th century lime and cement mortars: A Raman microspectroscopic study. *Mater. Charact.* **129**, 2–17 (2017).

S10. Edwards, H. G. M., Seaward, M. R. D., Attwood, S. J., Little, S. J., de Oliveira, L. F. C. & Tretiach, M., FT-Raman spectroscopy of lichens on dolomitic rocks: An assessment of metal oxalate formation. *Analyst* **128**, 1218–1221 (2003).

S11. Frost, R. L., Yang, J. & Ding, Z., Raman and FTIR spectroscopy of natural oxalates: Implications for the evidence of life on Mars. *Chinese Sci. Bull.* **48**, 1844–1852 (2003).
